# Light‐Guided Molecular Patterning for High‐Throughput Single‐Molecule Mechanical Characterization

**DOI:** 10.1002/smll.202511065

**Published:** 2026-01-19

**Authors:** Hansol Choi, Andrew Ward, Wesley P. Wong

**Affiliations:** ^1^ Program in Cellular and Molecular Medicine Boston Children's Hospital Boston Massachusetts USA; ^2^ Wyss Institute For Biologically Inspired Engineering Harvard University Boston Massachusetts USA; ^3^ Department of Biological Chemistry and Molecular Pharmacology Blavatnik Institute Harvard Medical School Boston Massachusetts USA; ^4^ Department of Pediatrics Harvard Medical School Boston Massachusetts USA

**Keywords:** bio‐lithography, biomaterials, biophysics, DNA nanotechnology, surface functionalization

## Abstract

Molecular patterning at single‐molecule resolution could significantly advance biomolecular analysis and the engineering of functional surfaces by enabling precise control over the spatial arrangement and mechanical accessibility of individual biomolecules. Such control is particularly valuable for multiplexed single‐molecule assays, which reveal molecular mechanisms, non‐equilibrium behavior, and nanoscale mechanical properties through the application of mechanical force. Standard surface functionalization methods, however, often lack sufficient precision, programmability, or accessibility, resulting in random or sparse biomolecular arrangements. To address this, we have developed a light‐guided surface patterning method that can covalently organize oligonucleotides (oligos) without the need for lithographic equipment. Oligos with 3‐cyanovinylcarbazole (CNVK) nucleoside are crosslinked in precise spatial arrangements defined by UV patterns projected through a digital micromirror device (DMD), with beads arranged accordingly. We demonstrate compatibility with established single‐molecule methods by performing single‐molecule force spectroscopy experiments on patterned coverslips, using magnetic tweezers and hydrodynamic‐based approaches. Our light‐guided method provides a scalable and accessible platform for biomolecular patterning that allows precise control over molecular identity and spatial positioning, enabling high‐throughput single‐molecule manipulation and mechanical characterization.

## Graphical abstract

A light‐guided molecular patterning method is presented for high‐throughput single‐molecule characterization. The approach enables scalable and accessible molecular patterning without specialized lithography equipment. Patterned surfaces are compatible with downstream biophysical assays, demonstrated by magnetic tweezers and flow‐based single‐molecule force spectroscopy, providing a versatile platform for probing molecular interactions.

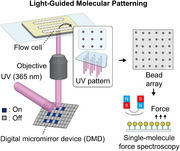

## 1 Introduction

The ability to precisely organize individual biomolecules on surfaces—down to the resolution of single molecules—presents powerful opportunities for biomolecular analysis, nanoscale engineering, and the design of advanced functional materials [1, 2, 3, 4, 5]. A particularly impactful application of such molecular‐scale patterning is single‐molecule force spectroscopy, a family of techniques that use mechanical force to probe nanoscale mechanical properties, molecular interactions, and biomolecular mechanisms [6, 7, 8, 9] including receptor binding [10, 11] and motor protein activity [12]. Although single‐molecule methods are providing valuable biological insights, studying biology “one molecule at a time” poses throughput challenges that can limit their broader application. To overcome this, multiplexed single‐molecule approaches have been developed, including parallel magnetic tweezers [13, 14], centrifuge force microscopy [15, 16, 17], acoustic force spectroscopy [18], and hydrodynamic force spectroscopy [19, 20, 21]. These methods apply precise mechanical forces to multiple molecules simultaneously, greatly increasing throughput by tracking numerous beads tethered to biomolecules on surfaces. Such multiplexed methods extend the capabilities of single‐molecule analysis, enabling studies of complex samples, molecular screening, and characterization of molecular heterogeneity. Importantly, the throughput and programmability of these multiplexed methods depend critically on surface functionalization [22]—the spatial arrangement and density of biomolecules and beads on the surface, as well as the specificity and robustness of linkage chemistries. Yet common functionalization strategies often produce either random and sparse distributions of beads, limiting throughput, or rely on expensive lithographic equipment (e.g. electron beam lithography), reducing accessibility. Furthermore, many existing methods depend on non‐covalent interactions (e.g., adsorption), restricting the magnitude of forces that can be applied during experiments [23].

Here, we present a photochemical surface‐patterning method that can precisely organize biomolecules at single‐molecule resolution without the need for photomasks or expensive lithographic equipment, enabling spatially resolved single‐molecule experiments on patterned substrates. Our approach uses oligonucleotides (oligos) modified by the photo‐crosslinkable nucleoside 3‐cyanovinylcarbazole (CNVK), which can form stable covalent attachments under spatially defined exposure to UV light (365 nm) [24, 25], with illumination of patterned UV achieved through the use of a digital micromirror device (DMD) [26, 27, 28]. To demonstrate programmability and precise spatial control, we create diverse biomolecular patterns including square and hexagonal lattices with tunable spacings. Moreover, oligos with distinct sequences can be sequentially patterned using a flow cell, enabling different molecular species to be precisely arranged for spatially resolved experiments. To demonstrate how this versatile and accessible patterning approach can facilitate highly parallel single‐molecule force spectroscopy, we perform parallel magnetic tweezers and hydrodynamic force spectroscopy experiments to stretch and unzip DNA constructs on multiplexed patterned surfaces. By integrating photochemical surface engineering with established single‐molecule techniques, our method opens new avenues for high‐throughput nanoscale mechanical characterization and biomolecular analysis.

## 2 Results and Discussion

### 2.1 Principles of Light‐Guided Molecular Patterning

**FIGURE 1:**
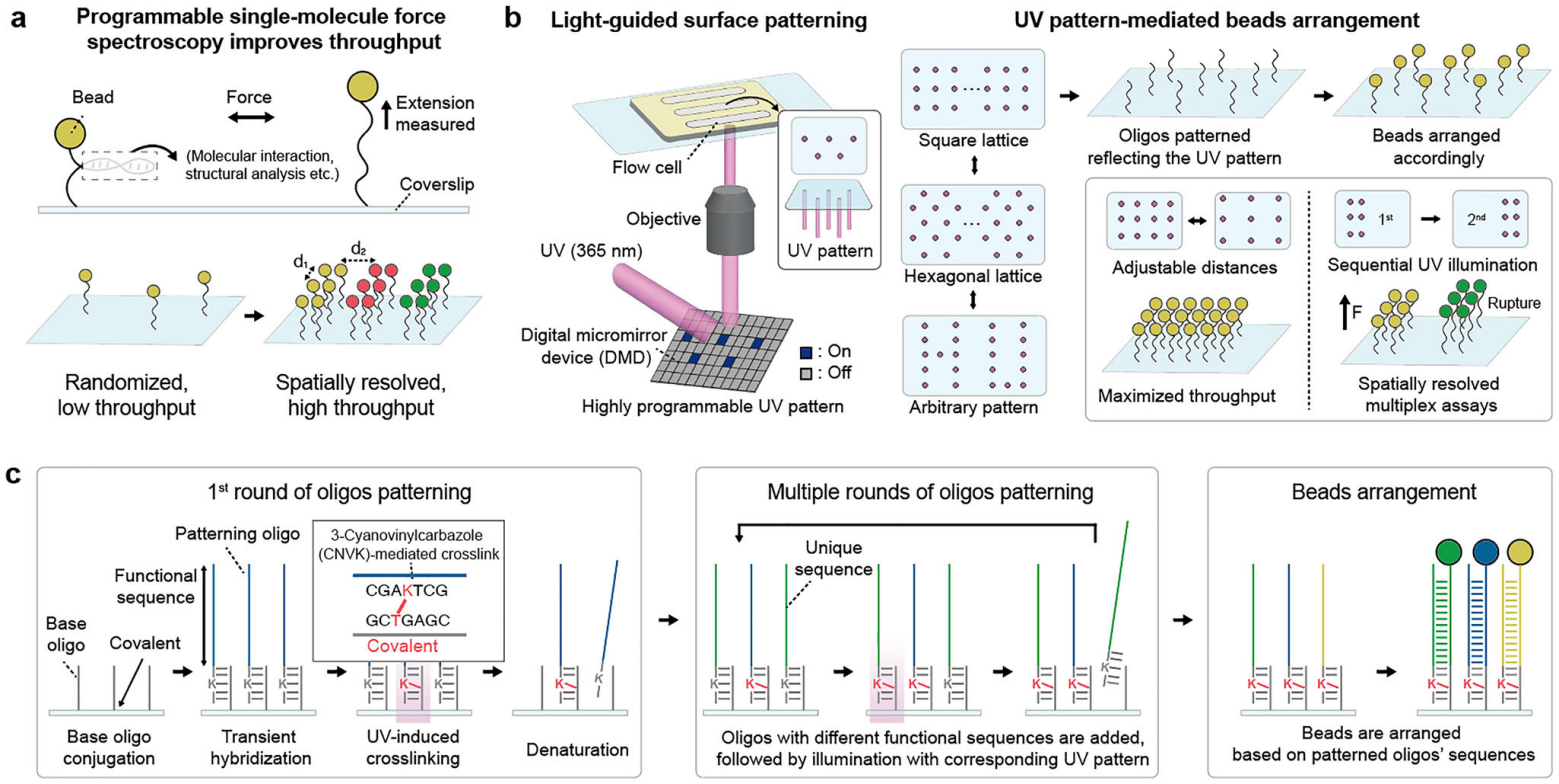
Light‐guided surface patterning enables spatially resolved multiplexed single‐molecule assays (a) The position of beads is precisely controlled to minimize interactions between adjacent beads and to enable multiplexed single‐molecule assays. (b) UV light is patterned through a digital micromirror device (DMD) to crosslink oligos where UV is illuminated. The distance between adjacent beads is programmable, allowing distinct oligos to be spatially resolved for multiplex experiments. (c) Base oligos are covalently conjugated to the surface, followed by hybridization with patterning oligos containing CNVK. Where UV is illuminated, patterning oligos and base oligos are crosslinked, and oligos that are not exposed to UV are denatured. This patterning process can be repeated to conjugate patterning oligos with different functional sequences. Beads are arranged based on the functional sequences of the crosslinked oligos.

Our light‐guided molecular patterning method enables the specific arrangement of oligos onto a solid surface; these oligos can furthermore be used to tether beads to targeted position for single‐molecule force studies (Figure 1a). Oligos are covalently attached to the surface via light‐activated CNVK crosslinkers, with their spatial arrangement determined by the illuminated UV pattern (Figure 1b). To passivate the surface and prepare it for patterning, 17 nucleotide (nt) base oligos modified with DBCO‐PEG13 are covalently conjugated to the azide‐functionalized coverslip using copper‐free click chemistry. Next, patterning oligos are added that can transiently hybridize to the base oligos (Figure S3). The patterning oligos consists of a 14 nt region, which includes a CNVK nucleoside, for hybridization to the base oligos, and a 55 nt functional region designed to capture DNA constructs for tethering beads. Upon UV illumination, the CNVK nucleosides crosslink to the thymine bases in the base oligos within seconds, enabling covalent oligos patterning. Transiently hybridized oligos that are not exposed to UV are denatured and washed with formamide to ensure oligo patterns match the UV pattern.

Regarding the patterning resolution, the nominal size of the illumination spot, not including diffraction, is approximately 390 nm by 390 nm when the UV is illuminated through a 20X objective with a single micromirror “pixel” activated. To experimentally characterize the spot size, taking into account the point‐spread function of our imaging/illumination system, fluorescent oligos complementary to the functional region were hybridized, and the fluorescence intensity of the resulting pattern was analyzed. The observed intensity was fitted to a Gaussian, resulting in a full width at half maximum (FWHM) of 992 nm (Figure S4).

To achieve sequence programmability, the flow cell can be patterned with multiple patterning oligos with unique functional sequences by repeating the cross‐linking and washing procedure multiple times, once for each sequence. This could be used to enable, for example, multiplexed spatially resolved single‐molecule force spectroscopy. The functional sequences of the patterning oligos were analyzed with NUPACK [29] to generate orthogonal sequences and minimize secondary structure.

To tether beads to the patterned oligos, DNA constructs that include a 55 nt region that is reverse complementary to these functional sequences are conjugated to streptavidin‐coated beads, which are then incubated with the surface (Figure 1c). Alternatively, constructs with biotin modifications can be hybridized to patterning oligos, followed by streptavidin‐coated bead incubation in the flow cell (Figure S5).

### 2.2 Fabrication of Programmable Bead Arrays

When performing single‐molecule force spectroscopy, the optimal distance between adjacent beads is determined by the lengths of the constructs connecting beads to the surface and the size of beads; this is particularly important with magnetic tweezers to prevent interactions between tethered beads [23]. While previous methods often require the creation of a photomask and/or the use of lithographic methods, which can be time consuming, our light‐based approach enables arbitrary patterns to be generated “on the fly.”

**FIGURE 2:**
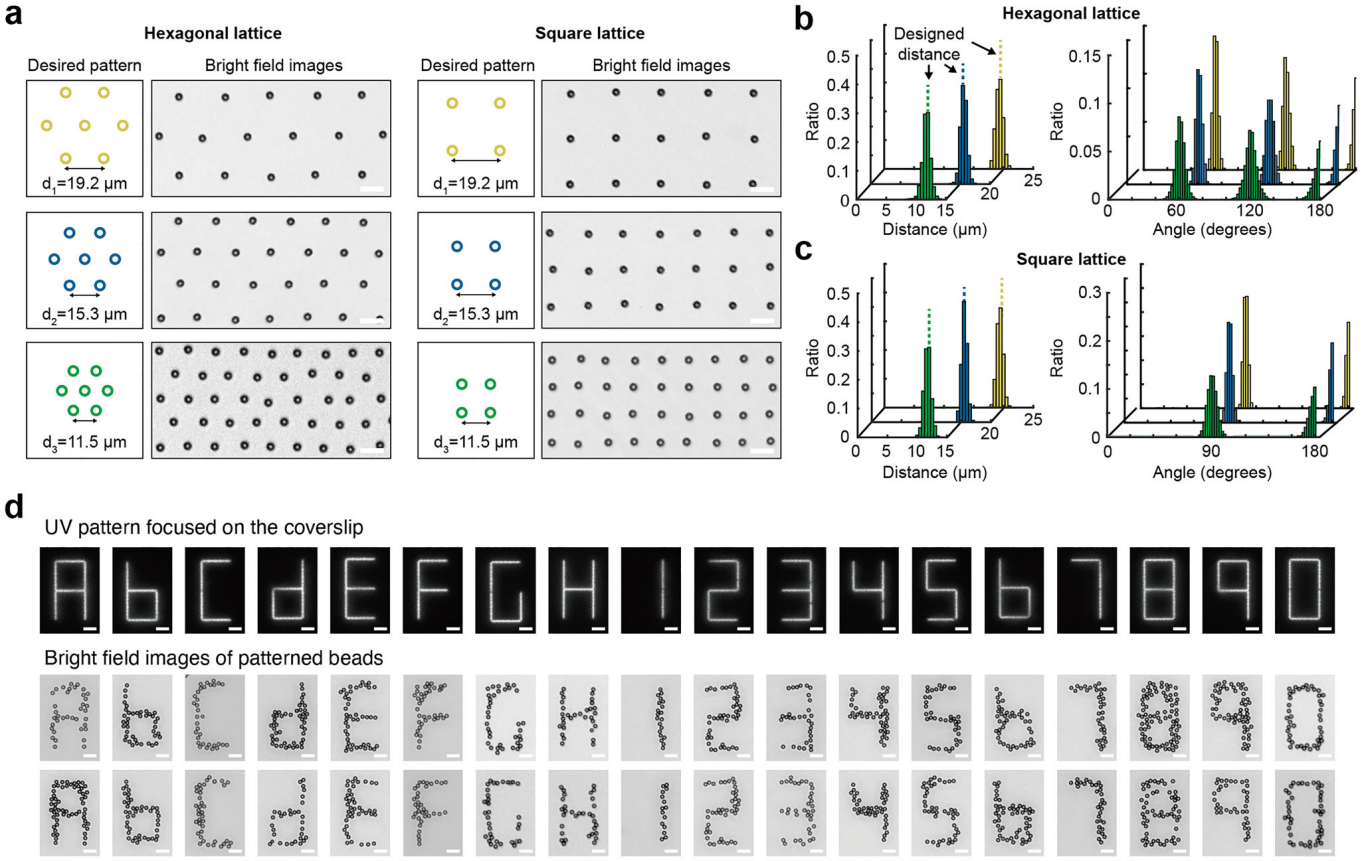
Diverse bead patterns enabled by a highly programmable patterning method. (a) Bright field microscopy images of beads organized into hexagonal and square lattice patterns with desired inter‐bead distances of 11.5, 15.3, and 19.2 µm. (b,c) Probability density distributions of inter‐bead distances and nearest neighbor angles for hexagonal lattice patterns (b) and square lattice patterns (c) are analyzed. For hexagonal lattices, distance *N* = 2923, 1549, and 829, and angle *N* = 13 173, 5833, and 2938 for lattice spacings of 11.5 µm (green), 15.3 µm (blue), 19.2 µm (yellow) respectively. For square lattices, distance *N* = 1175, 1096, and 511 and angle *N* = 4547, 2719, and 1939 for lattice spacings of 11.5 µm (green), 15.3 µm (blue), 19.2 µm (yellow) respectively. (d) Beads were arranged into 7‐segment geometries to demonstrate the arbitrary patterning capability. The top row shows the input UV illumination pattern focused on the coverslip, and the second and third rows show experimental replicates of resulting bead arrangements. Scale bar, 10 µm.

To demonstrate this flexibility and precise spatial positioning, beads arrays were fabricated in both hexagonal and square lattice patterns with three different bead spacings (Figure 2a). The primitive vector lengths or minimal distances between adjacent beads were set at 11.5, 15.3, and 19.2 µm for both square and hexagonal lattice patterns. The size of the illumination patch for each pattern was set to a nominal 780 nm by 780 nm square (2×2 pixels), sufficient to tether a single bead with a diameter of 2.8 µm on each patch and to prevent other beads from tethering due to steric hinderance. Streptavidin beads were conjugated with 55 nt biotin‐modified oligos that were reverse complementary to the functional sequence of patterning oligos on the flow cell surface. Subsequently, the oligo‐conjugated beads were introduced into the flow cell using a syringe pump with repeated cycles of infusion and withdrawal to increase the probability of tethering a bead to each spot (Figure S6). Untethered beads were washed away with buffer, and the remaining patterned beads were analyzed. Beads positions were measured using an edge detection algorithm employing the Sobel approximation [30] and three fields of view of the DMD were employed for the analysis of each condition (Figures S7–S12 and Note S1). The X and Y coordinates of the bead centers were measured to analyze distance and angle distributions (Figure S13). Although distances between all beads were calculated, a distance cut‐off of 1.3 times the length of the primitive vector was applied to specifically examine distances distribution between adjacent beads.

The mean and standard deviation (s.d.) of measured distances for the hexagonal bead lattices were 11.5 ± 0.67, 15.37 ± 0.62, and 19.07 ± 0.59 µm (mean ± s.d.), in excellent agreement with the designed distances (Figure 2b). Similarly, the square bead lattices exhibited distances of 11.49 ± 0.58, 15.31 ± 0.51, and 19.19 ± 1.17 µm (mean ± s.d.), also exhibiting good quantitative agreement (Figure 2c).

Angle distributions were analyzed by setting a reference bead as a vertex for each angle, with two additional adjacent beads employed to measure the angle between them. The same distance cut‐off was applied when selecting the two adjacent beads, and angle measurements were made for every bead as a vertex. All angle measurements gave the expected values. Specifically, for the hexagonal bead lattice with 11.5 µm spacing, measured angles were 60 ± 4.1°, 120 ± 5.1°, and 175.8 ± 3.6° while those with 15.3 µm showed 59.9 ± 3.7°, 120 ± 3.6°, 177.1 ± 2.5°, and those with 19.2 µm spacing showed 60 ± 2.1°, 120 ± 2.7°, 177.7 ± 1.8° (mean ± s.d.) respectively (Figure 2b). The angles measured from the square lattice bead arrays with a 11.5 µm spacing were 90 ± 4.1°, and 176 ± 3°. Similarly, angles measured from the square lattices with 15.3 and 19.2 µm spacings were 90 ± 2.5°, 177.7 ± 2.2°, and 89.6 ± 6°, 177.7 ± 3.6° (mean ± s.d.), respectively (Figure 2c).

As a further demonstration of the high spatial programmability of our patterning technology, various alphabets and numbers in seven segments, where each line segment had a length of 22.6 µm, were fabricated with beads (Figure 2d). In addition to the demonstrated patterns, any arbitrary patterns can be fabricated without any photomasks since the illumination pattern generated by the DMD can be immediately adjusted in response to an input image file.

### 2.3 Multiplexed Patterning with Oligos of Different Sequences

**FIGURE 3:**
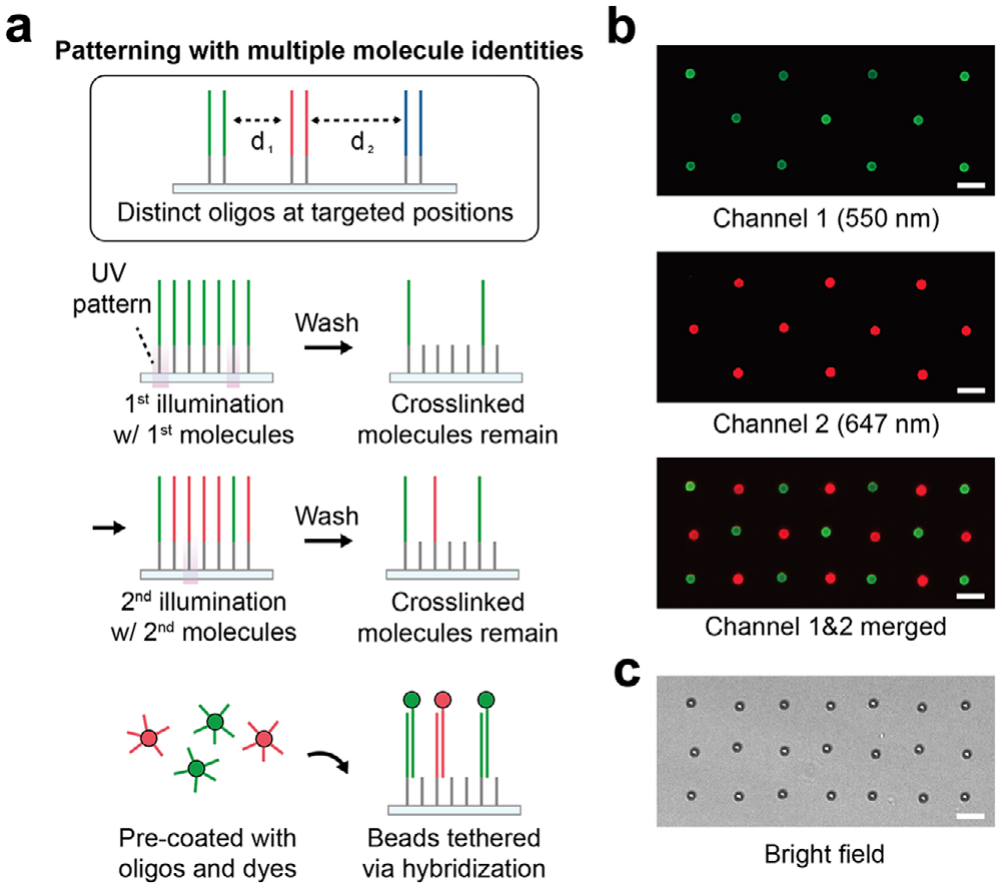
Spatially resolved multi‐sequence bead tethering by multiple rounds of patterning. (a) Two unique UV patterns are sequentially applied to conjugate two distinct patterning oligos. Beads covered with different dyes are attached to corresponding patterning oligos. (b) Fluorescence images acquired at 550 nm and 647 nm channels are shown alongside the merged image. (c) A bright field image of the same field of view is shown. Scale bar, 10 µm.

Our patterning method offers high molecular programmability, enabling the spatial organization of molecules with multiple distinct identities. By repeating the patterning process described above to create oligo patterns with distinct sequences, multiple different species of biomolecules or beads with different surface chemistries can be specifically and precisely attached to the surface (Figure 3a). We demonstrated this capability by patterning two distinct molecular species onto a surface. First, patterning oligos with sequence 1 were arranged on the surface in a square lattice pattern by illuminating the desired spots with UV light as before. This was then followed by a washing step and a second round of patterning. Specifically, a second type of patterning oligos with sequence 2 was introduced into the flow cell; during incubation, another square lattice UV pattern, shifted to illuminate the center of the unit cell of the initial lattice, was used to crosslink the second patterning oligos; this process was then followed by a stringent washing step to remove all non‐crosslinked oligos. Two different types of fluorescent beads were then tethered to these two distinct sequences. Beads were created by first incubating streptavidinated beads with biotinylated oligos that were reverse complementary to either functional sequence 1 or 2, followed by incubation with dyes with biotin or NHS‐ester modifications; a different dye was used for each type of bead. Incubation of these beads with the surface resulted in efficient tethering of beads to the lattice‐organized patterning oligos on the flow cell surface, with spatial organization based on sequence. After washing away the untethered beads, the resulting bead patterns were imaged with two distinct fluorescence channels (Figure 3b) and bright field (Figure 3c) to demonstrate the capability of our method to precisely position multiple distinct molecular targets (Figure S14).

### 2.4 Force Spectroscopy with Magnetic Tweezers on Patterned Surfaces

**FIGURE 4:**
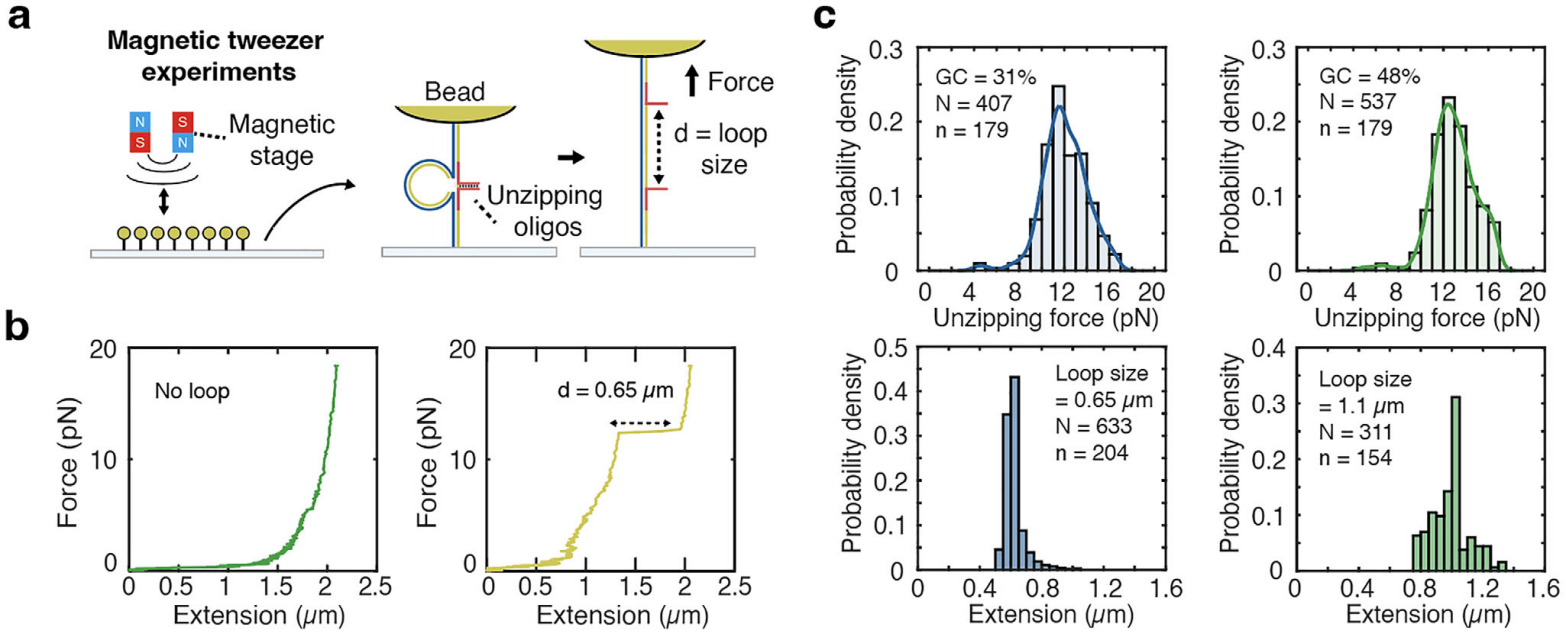
Magnetic tweezers experiments conducted on patterned coverslips. (a) Magnetic force was applied by lowering magnets to the sample on the patterned coverslip. DNA nanoswitch constructs with loop sizes of 1.1 and 0.65 µm, and unzipping oligos with different GC contents were designed. (b) Force‐extension curves of the DNA nanoswitch constructs. The left panel (green) shows the construct without unzipping oligos, and the right panel (yellow) show the construct with a loop size of 0.65 µm and 48% GC content. Dotted arrow depicts the change‐in‐extension at rupture/unbinding. (c) Histograms of unzipping force with GC content of 31% (top left), and 48% (top right) are shown. Histograms of extension of DNA nanoswitch due to unzipping with loop sizes of 0.65 µm (bottom left) and 1.1 µm (bottom right) are shown. For the construct with a GC content of 31%, *N* = 407 and *n* = 179; for the construct with a GC content of 48%, *N* = 537 and *n* = 179. For the construct with a loop size of 0.65 µm, N = 633 and n = 204; for the construct with a loop size of 1.1 µm, *N* = 311 and *n* = 154. The solid line represents the kernel density estimate (KDE), illustrating the estimated continuous probability distribution. *N* represents the total number of measurements collected across n molecules.

Single‐molecule force measurements with magnetic tweezers were conducted on the patterned flow cell. To facilitate repeated rupture measurements at single‐molecule resolution magnetic beads were tethered to the surface by DNA nanoswitches [16, 31, 32, 33], 7.2 kb long DNA constructs with two biomolecules of interest attached at different positions along the length [9, 21, 22] (Figure 4a). This construct forms a loop when these biomolecules bind to each other, and switches to a linear state when the bond breaks, resulting in an easily observable increase in length. For our demonstration, nanoswitches with two unzipping oligos designed to hybridized to different positions on the DNA construct with overhangs that were reverse complementary were used to measure the force‐driven DNA unzipping transition [34]. Four different types of DNA nanoswitches were created, with two different unzipping oligo sequences with GC contents of 48% and 31%, designed to measure different unzipping forces, and two different loop sizes of 1.1 and 0.65 µm, designed to produce different changes in length upon opening (Figure S15). The construct was prepared by linearizing circular single‐stranded DNA, followed by hybridization of 120 backbone oligos, each 60 nt in length to make the construct double‐stranded. The fabrication efficiency and the fraction of looped constructs were analyzed with gel electrophoresis, reaching up to 97.5% (Figures S16 and S17). The looping fraction varied depending on the unzipping oligo sequences and the loop size of the construct; unlooped constructs could result from unzipping oligos that fail to hybridize to the construct, from synthetic errors in the unzipping oligos [35] or from single‐stranded overhangs hybridizing with other unzipping oligos in the solution, thereby preventing loop formation.

For single molecule experiments, each spot would ideally be occupied by a single construct to decrease the chance of forming multiple tethers. To help facilitate this, a nominal illumination spot size of 390 nm was used, the smallest patch size achievable on our system with the 20X objective. Nanoswitch constructs with hybridization regions reverse complementary to the patterning oligos on one end and biotin‐modified oligos at the other end for coupling to magnetic beads were incubated in the flow cell. Given that the oligo density is gradually reduced over the steps of patterning oligo hybridization, UV exposure [36] and DNA nanoswitch construct hybridization (Figure S18), we optimized the number of single tethers on the patterned glass coverslip by controlling the UV power (Figure S19). Additionally, as discussed below, for single‐molecule force spectroscopy applications we validated single‐molecule tethers by using the distinct molecular signature of DNA nanoswitches.

The force on the molecular tethers was calibrated using the equipartition theorem [37, 38, 39] by measuring the mean square displacement of the beads at multiple different magnet heights (Note S2). At the start of each measurement cycle the DNA nanoswitch constructs were putatively looped and under low magnetic force; the force was then gradually increased up to 18.4 pN to unzip the construct and open the loop. Loop opening was marked by an abrupt change in extension that provided a clear molecular signature to characterize and verify the unzipping transition (Figure 4b; Figure S20). To filter out multiply tethered beads from those tethered with a single nanoswitch construct, we selected beads with tether lengths of at least 1.8 µm that exhibited a single loop‐opening event and loop sizes of at least 70% of the designed distances (1.1 or 0.65 µm) for further analysis, based on measurements obtained using magnetic tweezers, under which 83% of the analyzed beads passed the filtering criteria (Note S3 and Figure S2). DNA constructs with two different loop sizes were used, and a square lattice pattern with a minimal distance of 15.3 µm was used to prevent interaction between adjacent superparamagnetic beads during the application of magnetic force. The mean and standard deviation of unzipping forces for constructs with 48% GC content and loop sizes of 1.1 and 0.65 µm were 12.6 ± 2.2 and 13.1 ± 1.6 pN, respectively, with a mean loading rate of 30 ± 7 pN s^−1^ (mean ± s.d; Figure 4c, Data S1), showing unzipping forces are consistent regardless of the loop size. The unzipping forces for constructs with 31% GC content and loop sizes of 1.1 and 0.65 µm were 12.1 ± 2.1 and 12.0 ± 1.9 pN each with a mean loading rate of 27.1 ± 8.3 pN s^−1^ (mean ± s.d.). When the unzipping forces were calculated considering only the unzipping sequences, constructs with 31% GC content had a mean unzipping force of 12.0 ± 2.0 pN, and those with 48% GC content had a mean unzipping force of 12.9 ± 2.0 pN (mean ± s.d.). These measured mean forces and their GC‐dependent trend are broadly consistent with previous centrifuge force microscope (CFM) measurements under magnesium‐free conditions, once differences in loading rate are taken into account [16]. However, the rupture‐force distributions we observe here are broader, reducing the contrast between the 31% and 48% GC populations. We attribute this primarily to increased force heterogeneity inherent to our magnetic‐tweezers setup as compared to the extremely homogeneous force fields achievable with the CFM. Additionally, the extended lengths of DNA nanoswitches due to unzipping were 992 ± 119 nm for the constructs with a loop size of 1.1 µm, and 620 ± 63 nm (mean ± s.d.) for those with a loop size of 0.65 µm. The observed measurements were in good agreement with the expected values for dsDNA stretching under forces of 12 to 13 pN.

### 2.5 Hydrodynamic Force Spectroscopy on Patterned Surfaces

**FIGURE 5:**
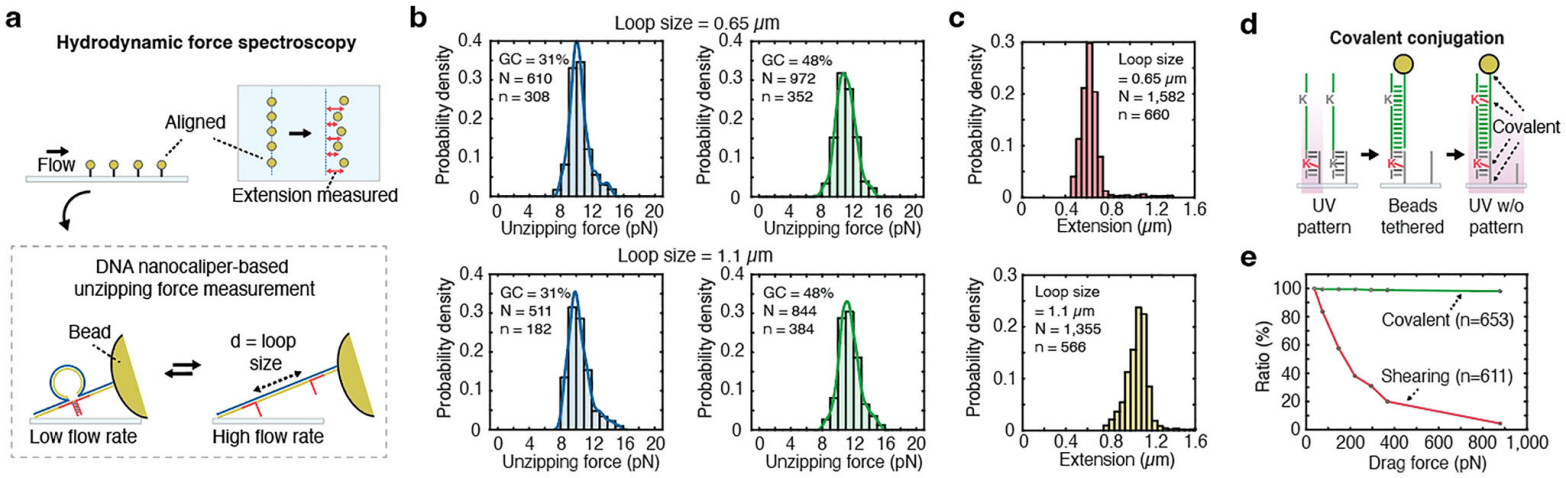
Hydrodynamic force spectroscopy conducted on patterned surfaces. (a) The positions of patterned beads are monitored during the application of hydrodynamic force. The same DNA nanoswitch constructs used in the magnetic tweezer experiments are employed. (b) Histograms of unzipping force with GC content of 31% and 48% measured using 0.65 µm and 1.1 µm loop‐sized constructs are shown. N represents the total number of measurements collected across n molecules. For the construct with a loop size of 0.65 µm and a GC content of 31%, *N* = 610 and *n* = 308; for the construct with a loop size of 0.65 µm and a GC content of 48%, *N* = 972 and *n* = 352. For the construct with a loop size of 1.1 µm and a GC content of 31%, *N* = 511 and *n* = 182; for the construct with a loop size of 1.1 µm and a GC content of 48%, *N* = 844 and *n* = 384. *N* represents the total number of measurements collected across n molecules. The solid line represents the kernel density estimate (KDE), illustrating the estimated continuous probability distribution. (c) Histograms of extension of DNA nanoswitch in X axis due to unzipping with loop sizes of 0.65 and 1.1 µm are shown. For the construct with a loop size of 0.65 µm, *N* = 1582 and *n* = 660; for the construct with a loop size of 1.1 µm, *N* = 1355 and *n* = 566. (d) Beads are patterned solely with covalent bonds by introducing two CNVK nucleosides in the patterning oligos. Following beads patterning by hybridization of functional region, UV is applied to the entire coverslip to covalently crosslink tethered beads. (e) The ratio of remaining beads on the patterned surface is measured by increasing the hydrodynamic force. The green curve represents putatively covalent tethers with UV applied after initial bead attachment. The red curve represents non‐covalent tethers with no UV applied after bead attachment; as these tethers are attached through DNA hybridization, they are susceptible to shearing off under the application of high levels of force.

To demonstrate that the method can be applied to various force spectroscopy experiments, single‐molecule force spectroscopy with hydrodynamic force was performed on the patterned flow cell (Figure 5a). The flow cell was connected to a syringe pump through polyetheretherketone (peek) tubing to apply hydrodynamic force based on the flow speed (Figure S6). The force calibration was conducted by calculating the drag force on the beads and the system's geometry, both of which were used to determine the tension applied to the construct [40] (Note S2 and Figure S1). The DNA nanoswitches used in the magnetic tweezer experiments were employed for hydrodynamic force spectroscopy, with beads tethered to each spot following the same protocol (Figure S21). To distinguish singly tethered beads from those with multiple tethers, we applied the same filtering criteria as in the magnetic tweezer experiments, determining tether lengths by comparing bead positions during flow infusion and withdrawal (Note S3). With a loading rate of 1.89 pN s^−1^ for DNA nanoswitches with a loop size of 0.65 µm, the unzipping forces of the unzipping oligos with 31% GC content were 10.3 ± 1.23 pN, while those with 48% GC content were 11.02 ± 1.22 pN (mean ± s.d.; Figure 5b and Data S2). For the constructs with a loop size of 1.1 µm, the loading rate was 1.99 pN s^−1^, and the unzipping forces of the unzipping oligos with 31% GC content were 10.35 ± 1.4 pN while those with 48% GC content were 11.39 ± 1.27 pN (mean ± s.d.). The ratio between the two unzipping forces were in good agreement regardless of the loop sizes. When the unzipping forces were calculated regardless of loop sizes, the ratio of unzipping forces between constructs with 31% GC content, measured using magnetic tweezers and flow experiments, was 1.16, while that for constructs with 48% GC content was 1.15, which is consistent with principles of dynamic force spectroscopy as lower loading rates generally decrease the value of the most probable rupture force [41, 42, 43]. Also, the nanoswitch length extensions along the X axis due to unzipping events were measured as 1060 ± 91 and 1102 ± 194 nm (mean ± s.d.) for constructs with a loop size of 1.1 µm and GC content of 48% and 31%, respectively. Similarly, extensions of 639 ± 127 and 635 ± 107 nm (mean ± s.d.) were observed for constructs with a loop size of 0.65 µm and GC content of 48% and 31%, showing that the extensions are consistent regardless of the unzipping oligos’ sequences (Figure 5c).

### 2.6 Bead Patterning with Covalent Bonds for High Force Application

Hydrodynamic force spectroscopy enables a relatively wide range of forces to be applied to single molecules, with a higher maximum force than typical magnetic tweezers systems. However, the maximum usable force is limited by the strength of the interactions tethering the beads to the surface—in particular, non‐covalent interactions may simply not survive. To address this challenge, we adapted our patterning method to tether beads covalently by introducing two CNVK nucleosides to the patterning oligos (Figure 5d). The bead patterning procedure is essentially the same as before, except for the introduction of a CNVK nucleoside in the functional sequence of the patterning oligo, and the usage of azide‐coated magnetic beads. The beads with a diameter of 3.06 µm and DBCO‐modified oligos were incubated to functionalize beads with oligos through copper‐free click chemistry, and then functionalized beads were incubated onto the patterned flow cell to arrange them through oligo hybridization. Following beads arrangement, UV was illuminated through a handheld UV lamp to the whole flow cell to covalently crosslink the oligos patterned on the flow cell with those functionalized on the beads via CNVK. Beads were organized in a square lattice pattern with 15.3 µm spacing, and the ratio of remaining beads under distinct hydrodynamic force application was investigated.

To evaluate the effectiveness of our covalent tethering approach, we performed hydrodynamic force spectroscopy experiments at high forces, comparing the covalently patterned surfaces to the non‐covalently patterned surfaces. We applied the flow to both surfaces as a step function with 7 distinct flow rates, increasing sequentially from low to high, each maintained for 5 s. Drag force applied to beads is calculated by considering the bead size and shear rate (Note S2). With the non‐covalent approach, less than 40% of the tethered beads remained after application of a drag force of 220 pN per bead, corresponding to a flow rate of 300 µL min^−1^ (Figure 5e). In contrast, the beads that were tethered covalently survived much higher forces, with 98% of the beads (*n* = 653) remaining even under application of a maximum drag force of 880 pN per bead, corresponding to a flow rate of 1,200 µL min^−1^.

## 3 Conclusion

In this article, we presented a light‐guided molecular patterning method capable of precisely arranging biomolecules with multiple identities and beads in arbitrary patterns without the need for expensive lithographic equipment. As a demonstration, oligos with unique sequences were precisely positioned and identified by tethering them to beads coated with distinct fluorophores. As an application, we showed how our method enables multiplexed single‐molecule force spectroscopy measurements, carrying out bond rupture experiments with both magnetic force and hydrodynamic force on patterned flow cells. By combining this assay with nanoengineered DNA nanoswitch constructs, we successfully measured the unzipping forces of oligos of different sequences, obtaining mean forces and GC‐dependent trends that align well with previous measurements. At the same time, our data highlight that bead‐to‐bead variability and spatial variations in the applied force field can broaden rupture‐force distributions and reduce contrast when analyzing individual rupture events. We anticipate that incorporating per‐bead force calibration and extensive per‐molecule averaging would significantly sharpen these distributions and further improve measurement precision. Uniquely, our molecular patterning strategy allows single molecules to be covalently tethered to surfaces with high spatial accuracy, making it compatible with a broad range of force spectroscopy techniques and enabling multiplexed, high‐throughput experiments in which repeated force measurements on the same molecules can be used to resolve molecular heterogeneity with greater precision.

For comparison, previous approaches have demonstrated the ability to track multiple molecules by randomly positioning them on surfaces, either through physisorption with nitrocellulose [16] or via covalent interactions, such as N‐Hydroxysuccinimide (NHS) ester conjugation or click chemistry [44]. However, the lack of precise control over spatial positioning can limit throughput and the ability to perform spatially resolved experiments with multiple molecular identities. More recently, a soft lithography‐based approach has been proposed to pattern the surfaces with proteins, enabling the precise arrangement of oligos using patterned substrates such as a polydimethylsiloxane (PDMS) stamp [23]. While this approach is effective in increasing throughput and enabling adsorption or covalent immobilization [45, 46], it requires a master mold to be prepared through photolithography for each pattern, limiting accessibility and making it a challenge to generate patterns with multiple molecular identities In contrast, our light‐guided approach enables fabrication of arbitrary molecular patterns with diverse molecular identities by simply manipulating a DMD within a few seconds, and is well‐suited for high force application. Moreover, crosslinked CNVK nucleoside can be split under irradiation of 312 nm UV [24], allowing for potential resetting or reusing of specific regions of the flow cell multiple times. Our method utilizes a commercially available DMD (Polygon 400, Mightex Inc.) that can be easily attached to microscope, and it can be used to pattern biomolecules through conjugation to oligos for various purposes such as proteins for cell patterning and nanoparticles for photonics applications.

Our light‐guided surface‐patterning approach enhances the throughput of biomolecular assays by enabling the rapid, precise, and programmable arrangement of multiple molecular species, thereby increasing the number of simultaneously trackable molecules—a significant improvement for single‐molecule studies. Due to its programmability, versatility—facilitating both covalent and non‐covalent attachments—and its speed, scalability, and accessibility, we anticipate this technology will broadly impact diverse research areas including biophysics, nanotechnology, molecular diagnostics, biophotonics, and advanced surface engineering.

## 4 Experimental Section

### 4.1 Oligo Design and DNA Nanoswitch Construct Preparation

The 3’ end of the 17 nt base oligo is modified with DBCO‐PEG13 (Polyethylene glycol linker) to attach it to the azide coverslip (104‐00‐626, PolyAn) and passivate the surface. Each patterning oligo consists of a 55 nt construct hybridization region as well as a 14 nt base oligo hybridization region that includes a CNVK nucleoside to crosslink it upon UV illumination (Figure S2 and Table S1). The DNA nanoswitch construct for force spectroscopy is synthesized with circular M13mp18 ssDNA (N4040S, New England Biolabs). To linearize the circular DNA, 10 µL of 110 nm M13mp18 ssDNA, 7 µL of 10 µm 20 nt of cutting strand (Table S1) that hybridizes to the cutting region in circular DNA, and 2 µL of 10X rCutSmart buffer were annealed for 20 min by heating to 90°C and slowly cooling down to 50°C at a rate of 30 s per °C. Next, 1 µL of BtsCI restriction enzyme (R0647S, New England Biolabs) is added followed by incubation at 50°C for an hour and inactivation at 80°C for 20 min. To fabricate the dsDNA linear construct, linearized M13mp19 ssDNA at 1 nm concentration was incubated with 5 nm of backbone oligos 1–119 (Data S3), and 2 nm of backbone‐biotin oligo by heating to 90°C and slowly cooling down to 80°C at a rate of 1 min per °C and 80°C to 20°C at a rate of 6 min per °C. To fabricate the construct with a loop size of 1.1 µm, backbone oligos 116 and 117 were replaced with unzipping oligos with overhangs (Table S1). To fabricate the construct with a 0.65 µm loop, backbone oligos 118 and 119 were replaced. Fabricated constructs were analyzed with 0.7% agarose gel pre‐stained with SYBR safe (S33102, Invitrogen), which was run at 100 V for around 2 h in a 4 C room. Base oligo with DBCO‐PEG13 modification and patterning oligos with CNVK modification were purchased from Gene Link and other oligos were purchased from IDT (Integrated DNA Technologies) unless otherwise specified.

### 4.2 Flow Cell Preparation

Buffer A (PBS, pH 7.4), buffer B (PBS, 1% Tween 20, pH 7.4), buffer C (PBS, 1% Tween 20, 500 mm NaCl, 0.01% (w/v) blocking reagent; 11096176001, Roche), buffer D (PBS, 1% Tween 20, 0.01% (w/v) blocking reagent), and blocking buffer (PBS, 1% Tween 20, 1% (w/v) blocking reagent) were prepared for the experiments. Flow cells were fabricated by sandwiching glass slides (48300‐037, VWR International) and azide‐coated coverslips with double‐sided tape [47]. A flow chamber with channel dimensions of 1.96 mm width, 26.46 mm length, and 100 µm height was created by cutting double‐sided tape using a cutter plotter (CE7000‐60, Graphtec), prior to assembly. Glass slides were drilled using a diamond drill bit with a diameter of 0.75 mm (415601, SFJS) to make inlets and outlets. The channel was incubated with 100 µm of DBCO‐PEG13 modified base oligo in buffer A for 18 h. After washing the channel three times with buffer B, the channel was incubated with the blocking buffer for 30 min twice followed by washing with buffer C. 100 nm of patterning oligo in buffer C was incubated for 10 min and the patterned UV was illuminated. Patterning oligos that are not crosslinked to the base oligos are washed away by incubating with 40% formamide for 3 min followed by washing with buffer D. Flow cells were stored with buffer D at 4°C or within a vacuum chamber and were incubated with blocking buffer for an hour before each experiment.

### 4.3 Oligo Patterning Using a DMD

The desired pattern of 365 nm UV was illuminated following the previously reported method [26]. Briefly, 365 nm UV (BLS‐Series 50 W emitter, Mightex) was illuminated through a 20X objective with numerical aperture (NA) of 0.5 (CFI Plan Fluor, Nikon) at a power setting of 4% for 2 s. The pattern was adjusted using the Nikon Elements Polygon 400 module interface by uploading a bitmap file, where the software maps each pixel to a corresponding micromirror centering the bitmap file on the micromirror array. The system is compatible not only with the setup described here, but also with other microscope platforms, and can alternatively be implemented with a custom DMD and light guide [48, 49]. UV power can be adjusted based on the number of on‐target beads, and the number of singly tethered beads as increased power will increase the crosslinking efficiency [36].

### 4.4 Bead Conjugation to Each Patterned Spot

M270 Dynabeads (65305, ThermoFisher Scientific) were used for magnetic tweezers and hydrodynamic force spectroscopy experiments. To prepare the beads for experiments of non‐covalently attached beads, 10 µL of 10 mg mL^−1^ beads were washed with 1X Binding and Wash (B&W) buffer (50 mm sodium phosphate, 300 mm NaCl, and 1% Tween20) for three times followed by incubation with 100 µL of 10 nm biotinylated functional oligo in 1X B&W buffer (Table S1). Next, the beads were washed with buffer B three times, and the surface of the beads was blocked by incubation with 50 µL of blocking buffer for 1 h. Beads were washed with buffer B before usage. The flow cell and 1 mL gastight syringe (1001‐TLL, Hamilton Company) were connected through peek tubing (1569, P628, F‐247, and XP‐235, IDEX Corporation), and beads were added to the flow cell at a flow rate of 20 µL min^−1^ using a syringe pump (70‐4504, Harvard Apparatus). To functionalize the surface of the flow cell, 1 nm of DNA nanoswitch construct was incubated with the flow cell for 3 h at room temperature followed by wash and bead incubation steps. For the covalent conjugation of beads, azide magnetic beads (CBPMC01c, Bangs Laboratory Inc.) and 55 nt DBCO‐modified functional oligo were used. Washed azide beads (100 µg) were incubated with 100 µL of 100 nm DBCO oligos for 2 h. Beads were washed with buffer B three times and incubated with blocking buffer for an hour before usage. After bead assembly through hybridization of the functional sequence, the flow cell was carefully moved to the top of a handheld 6 W UV lamp (97620‐22, Cole‐Parmer) and illuminated with 365 nm UV light for 30 min.

### 4.5 Statistical Analysis

Force and extension results from magnetic tweezer experiments and hydrodynamic force spectroscopy with multiple tethers were filtered out based on the quantitative criteria described in Note S3. Mean and standard deviation (mean ± s.d.) were reported where applicable. Sample size N represents the total number of measurements collected across n molecules, as stated in the figure legends.

## Author Contributions

H.C. and W.P.W. conceptualized and designed the experiments. H.C. and A.W. conducted single‐molecule force spectroscopy experiments. H.C., A.W., and W.P.W. participated in data analysis and discussion. H.C. and W.P.W wrote the original draft and edited the manuscript.

## Funding

This work was supported by the National Institute of General Medical Sciences (NIGMS; R35GM119537) (W.P.W.) and the International Human Frontier Science Program Organization (LT0005/2023‐C) (H.C.).

## Conflicts of Interest

The authors declare no conflicts of interest.

## Supporting information




**Supporting file 1**: smll72433‐sup‐0001‐SuppMat.pdf

**Supporting file 2**: smll72433‐sup‐0002‐DataFile.zip

## Data Availability

All data are available in the main text or the supporting information.
