# Supplementary material for: Light‐Guided Molecular Patterning for High‐Throughput Single‐Molecule Mechanical Characterization
Source: Small. 2026 Jan 19;22(21):e11065. doi: 10.1002/smll.202511065 (PMC13081101; doi:10.1002/smll.202511065)
Supplement: Supplementary file 1 — Supporting file 1: smll72433‐sup‐0001‐SuppMat.pdf [file SMLL-22-e11065-s001.pdf]

Supporting Information for

Light-Guided Molecular Patterning for High-Throughput Single-Molecule Mechanical Characterization

*Hansol Choi, Andrew Ward, Wesley P. Wong**

*Correspondence should be addressed to W.P.W. (wesley.wong@childrens.harvard.edu)

This file includes:

Note S1 – S3

Figure S1 – S21

Table S1

Note S1 MATLAB Code for beads detection

For reproducibility, a MATLAB code that are used to detect beads from microscope images is provided.

```
------------------------------------------------------------------------
close all; clear all;

% Read the TIF image
img = imread('image_file.tif');

% Adjust based on spacings
dis_threshold = 55;

% Microscope pixel size
um_per_px=0.3984;

img = im2double(img);

% Edge detection
edge_img = edge(img, 'Sobel');

% Dilation
se = strel('disk', 3); % Adjust the size based on the particle size
binary_img = imdilate(edge_img, se);

% Connected component analysis
cc = bwconncomp(binary_img);
stats = regionprops(cc, 'Centroid', 'Area');

centroids_single = [];

% Visualize the detected particles
figure;
img=imadjust(img, [0.05 0.3]);

imshow(img)
hold on;

% Areal threshold to detect only single particles
area_threshold = 180;

% Plot the detected particles
for i = 1:numel(stats)
   centroid = stats(i).Centroid;

   if stats(i).Area <= area_threshold
      centroids_single = [centroids_single; centroid];
      plot(centroid(1), centroid(2), 'g*', 'Markersize', 5);
   end
end

% Acquire angle & distance
num_particles_single = size(centroids_single, 1);
```

```
all_distances = [];
all_angles = [];

% Measure distance
for i = 1:num_particles_single-1
    for j = i+1:num_particles_single
        distance_ij = sqrt((centroids_single(i, 1) - centroids_single(j, 1))^2 + (centroids_single(i, 2) -
centroids_single(j, 2))^2);
        if distance_ij <= dis_threshold
            all_distances = [all_distances,distance_ij];
        end
    end
end


% Measure angle
for i = 1:num_particles_single
    for j = 1:num_particles_single
        distance_ij = sqrt((centroids_single(i, 1) - centroids_single(j, 1))^2 + (centroids_single(i, 2) -
centroids_single(j, 2))^2);

        if distance_ij <= dis_threshold && i ~= j

            for k = 1:num_particles_single
                distance_ik = sqrt((centroids_single(i, 1) - centroids_single(k, 1))^2 + (centroids_single(i, 2) -
centroids_single(k, 2))^2);

                if distance_ik <= dis_threshold && i ~= k && j < k
                    vji = centroids_single(j, :) - centroids_single(i, :);
                    vki = centroids_single(k, :) - centroids_single(i, :);

                    angle_degrees = acosd(dot(vji, vki) / (norm(vji) * norm(vki)));
                    all_angles = [all_angles, angle_degrees];


                end
            end
        end
    end
end

% Plot the histogram
figure;
histogram(all_distances, 'BinEdges', 1:1:100, 'Normalization', 'probability');
hist_counts_dis = histcounts(all_distances, 'BinEdges', 1:1:100);

figure;
histogram(all_angles, 'BinEdges', 0:2:180, 'Normalization', 'probability');
hist_counts_angle = histcounts(all_angles, 'BinEdges', 0:2:180);
```

Note S2. Magnetic tweezers and flow cell force calibration & experiments

Magnetic force applied to beads was calculated using the equipartition theorem[1–3] where $F = \frac{k_B T L}{<X^2>}$, k_B is the Boltzmann constant, T is absolute temperature, L is extension of the tether, and $<X^2>$ is the mean square displacement from the mean position along the x-axis, which was parallel to magnetic field. To determine the force calibration curve for different magnet heights, bar magnets with a 1 mm gap were placed at a variety of different Z positions (0.5 mm, 1 mm, 2 mm, and 5 mm) from the glass slide while L and $<X^2>$ were measured[4]. The distance between the coverslip surface and the top side of the glass slide was 1.3 mm since the channel height was 0.1 mm and the glass thickness was 1.2 mm. To interpolate between different magnet positions, the force vs magnet position data was fitted to an exponential curve. To measure DNA unzipping events, the magnet was moved from a position of 10 mm to a position of 0.3 mm with a magnet speed of 1 mm/s; images were taken with a frame rate of 50 frame per second (fps). For the magnetic tweezer experiments, a 60X objective with an NA of 1.27 (Plan Apo IR, water immersion, Nikon) was used.

For the calibration of the hydrodynamic force under flow, we calculated the geometry of the system and the tension on the DNA construct at different flow rates using a previously reported method[5] based on fluid dynamics theory. Using force balance and torque balance and the hydrodynamics of laminar flow near a surface, one can derive Equations 1 and 2, where a = 1.7, the ratio of torque to drag force R = 0.37r, with r as the bead radius, η is the flow viscosity, which we set to $\eta = 10^{-3} Ns/m^2$, F_T is the tension, F_{drag} is the drag force applied to the bead, v is the flow velocity, α is the tether angle relative to the coverslip, and κ is the angle between the imaginary line connecting the point where the bead touches the surface and the DNA-bead attachment point, and the vertical line passing through the bead's center (Figure S1). The flow velocity v was calculated as $r\gamma$, where the shear rate $\gamma = \frac{6Q}{wh^2}$, Here Q is the volumetric flow rate, w = 1.9 mm is the channel width, and h = 100 μm is the channel height, which simplifies the equation to v (μm/s) = 7.15Q (μl/min) when 2.8 μm beads are used. Equation 1, and 2 are derived from force and torque balance, while equation 3 relating $x, \alpha, and\ \kappa$ is derived from the geometry of the system (Figure S1). Equation 4, which relates x and α is obtained by combining Equations 1–3. Additionally, the bead center to center distance (D_{cc}) during flow infusion and withdrawal is experimentally measured, and Equation 5 is derived based on the system's geometry. To measure D_{cc} for the DNA nanoswitch construct in the looped conformation, we supplemented the buffer with 20 mM of $MgCl_2$ to increase the unzipping force and used higher

loading rates. The flow infusion rate was ramped from 0 µl/min to 50 µl/min for 5 seconds, followed by a flow withdrawal at a constant 50 µl/min flow rate. The D_{cc} was calculated under the assumption that the extension profile of a single construct should be symmetric between flow infusion and withdrawal.

$$Force\ balance: F_T cos(\alpha) = F_{drag} = a6\pi\eta rv \quad (1)$$

$$Torque\ balance: F_T cos(2\kappa - \alpha) = F_{drag} R \quad (2)$$

$$From\ geometry: xsin(\alpha) = r\big(1 - cos(\pi - 2\kappa)\big) \quad (3)$$

$$\tan(\alpha) = \frac{1 + R - \frac{xsin(\alpha)}{r}}{\sqrt{(\frac{xsin(\alpha)}{r})^2 + \frac{2xsin(\alpha)}{r}}} \quad (4)$$

$$From\ geometry: D_{cc} = 2(x(cos(\alpha) + rsin(2\kappa)) \quad (5)$$

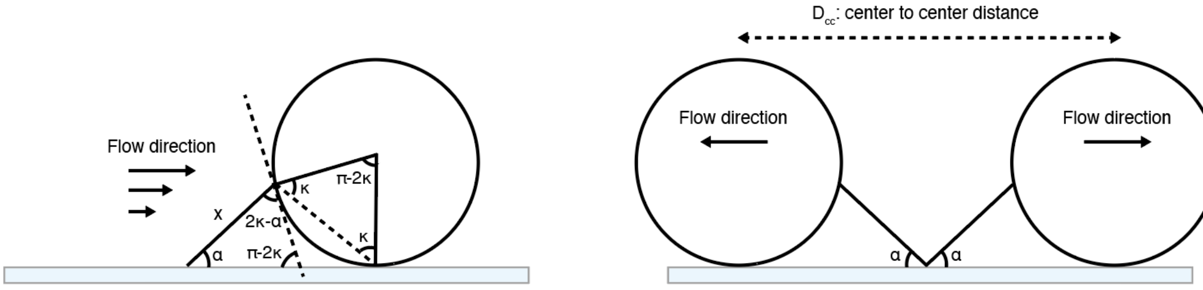

Figure S1. Geometry of a bead tethered to a DNA construct under flow conditions[5].

As most of the unzipping was observed between the flow rate of 25 µl/min and 30 µl/min, we calculated the angles and x by numerically solving the equations at these two flow rates using MATLAB. For the constructs with a loop size of 0.65 µm, we found $\alpha = 32.71°, \kappa = 57.34°,\ x = 1.51\ \mu m, and\ F_T = 9.53\ pN$ when the flow rate was 25 µl/min and $\alpha = 32.59°, \kappa = 57.28°,\ x = 1.52\ \mu m, and\ F_T = 11.4\ pN$ when the flow rate was 30 µl/min. For the constructs with a loop size of 1.1 µm, we found $\alpha = 36.45°, \kappa = 59.27°,\ x = 1.23\ \mu m,\ and\ F_T = 9.97\ pN$ when the flow rate was 25 µl/min and $\alpha = 36.45°, \kappa = 59.27°, x = 1.23\ \mu m, and\ F_T = 12\ pN$ when the flow rate was 30 µl/min. Next, to interpolate the force as a function of flow rate, we assumed that the force is linearly correlated to the flow velocity within this range of flow rates as the calculated change in angle was small between the flow rates of 25 µl/min and 30 µl/min, and since $F_T = \frac{F_{drag}}{\cos(\alpha)}$ where drag force is proportional to flow velocity.

Note S3. Validation of single-molecule tethers using DNA nanoswitches

Reliable single-molecule force spectroscopy requires clear identification of beads attached by single-molecule tethers. Our approach leverages engineered molecular constructs that provide *single-molecule signatures* to reliably distinguish single tethers from multiple tethers or non-specific interactions. Specifically, we use DNA nanoswitches, self-assembled mechanical switches that we developed using structural DNA nanotechnology, which provide a distinct force-extension signature for single-molecule identification, and a durable linkage between interacting pairs of molecules to enable the repeated interrogation of their interaction. Each DNA nanoswitch consists of 7.2 kb double-stranded DNA scaffold to which molecules of interest can be precisely and specifically linked through DNA hybridization. Furthermore, by attaching anchoring molecules to its two ends (e.g. biotin and/or digoxigenin), the nanoswitch can serve as a tether between a functionalized bead and a functionalized surface. When two molecules on the DNA nanoswitch interact with each other (e.g., DNA hybridization or protein–protein binding), the nanoswitch forms a loop, shortening its end-to-end extension by a known, design-defined amount. This generates a unique mechanical signature (force-extension profile) when pulling on the tethered bead, enabling us to unambiguously distinguish single-molecule tethers from multiple tethers and nonspecific interactions, and to determine the state of the attached molecules (bound/unbound).

During force spectroscopy experiments, we record force-extension traces, i.e. we measure the DNA end-to-end distance while gradually increasing the force applied to the molecules. As the force is increased the extension asymptotically approaches the contour length of the DNA under tension. When the interacting molecules on the nanoswitch are not bound together, we observe a contour length corresponding to the full 7.2 kb construct. When they are bound together, the nanoswitch is looped, and the observed total extension reflects this shorter tether length; rupture/unbinding between these interacting molecules results in a sudden change-in-extension that reflects the size of the loop. For singly tethered beads, the measured values should match the designed construct dimensions.

In contrast, beads with multiple tethers deviate significantly from this behavior. For example, they may show a smaller total extension than expected, because a single bead connected to the surface by multiple tethers oriented at different angles will be more constrained than a singly tethered bead. Similarly, both the rupture force and the change-in-extension at rupture may vary from the expected values, since the force applied to the bead is distributed among the

multiple DNA nanoswitch tethers aligned along the force direction. Additionally, multiple tethers often rupture at different forces, resulting in a characteristic force-extension trace with multiple changes-in-extension.

To systematically verify single-molecule tethering, we apply the following quantitative criteria:

1. Constructs with an end-to-end distance after rupture (e.g., DNA unzipping) smaller than 1.8 μm are excluded, as the designed length at this expected force level is ~2.3 μm (Figure S13). We also note that beads that are stuck to the surface or stuck to other beads are excluded from analysis.

2. The change-in-extension due to rupture must exceed 70% of the designed loop size. For example, the cutoff was 0.77 μm for constructs with a loop size of 1.1 μm, and 0.46 μm for those with a loop size of 0.65 μm.

3. Results showing more or less than one rupture event were excluded.

Only beads whose force-extension traces satisfy all three criteria are classified as singly tethered and included in the single-molecule force analysis, accounting for over 83% of analyzed beads.

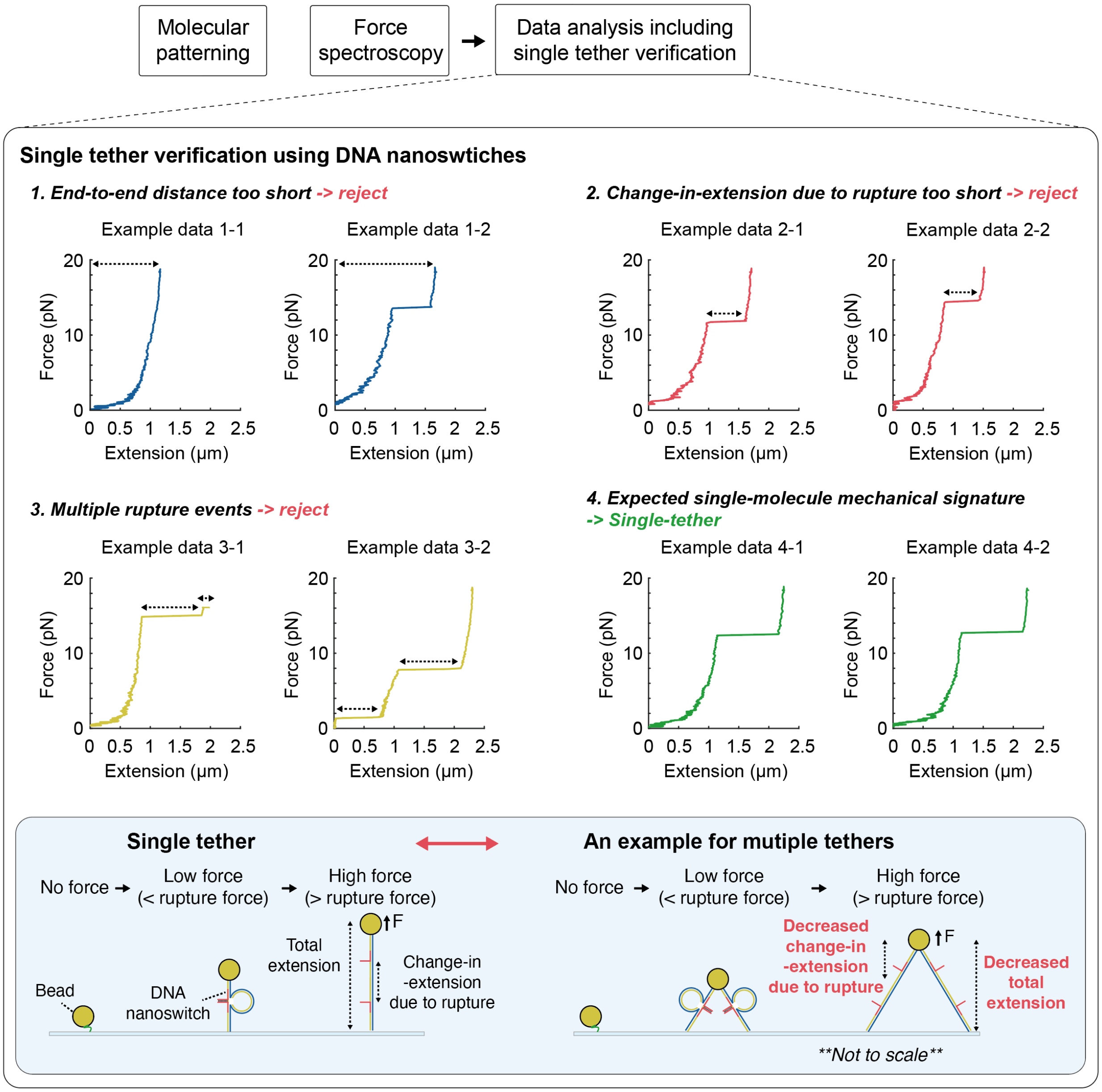

Figure S2. Single-tether verification using DNA nanoswitches. (Top) Outline of workflow for taking and analyzing single-molecule force spectroscopy data. (Middle) DNA nanoswitches generate molecular signatures that enable the identification of singly tethered molecules. Graphs represent example force-extension traces acquired using DNA nanoswitches that demonstrate the filtering process. (Bottom) Cartoon schematic comparing the mechanical signatures of beads attached via a single tether (left) versus multiple tether (right). All example data shown correspond to DNA nanoswitches with a loop size of 1.1 μm and unzipping oligos with 48% GC content.

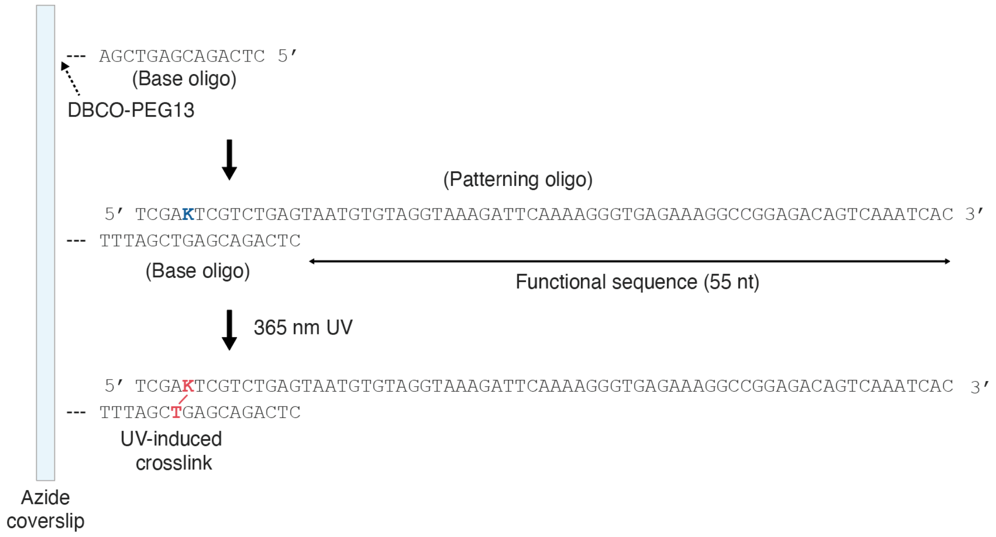

Figure S3. Base oligo and patterning oligo sequence design.

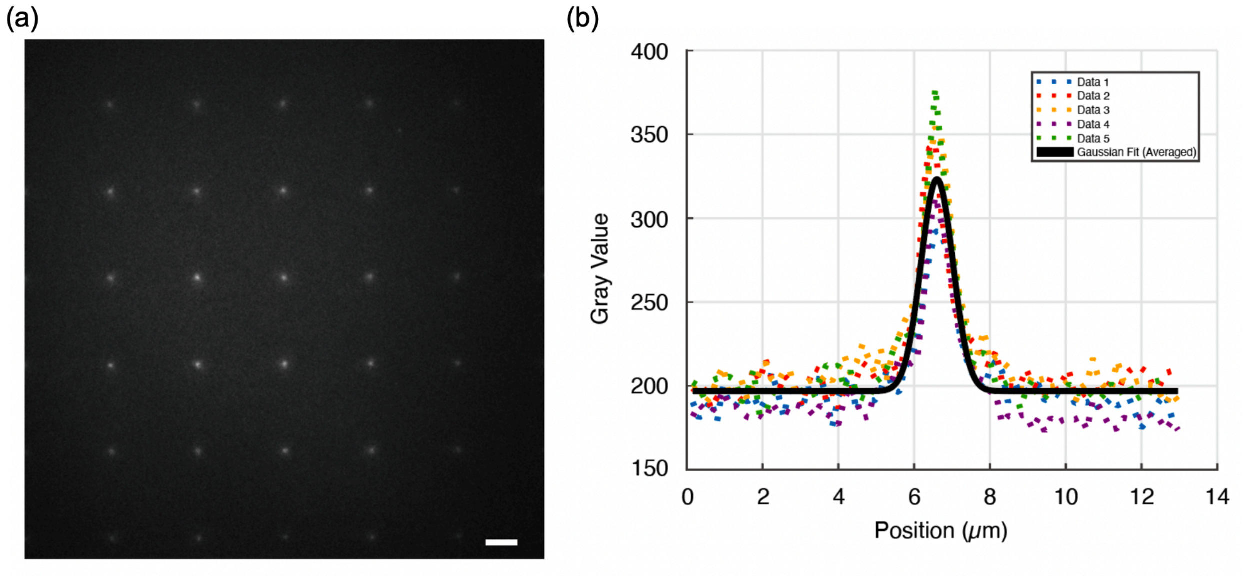

Figure S4. A square array in which each spot was patterned using a single micromirror was characterized by hybridizing fluorescent oligos complementary to the functional sequence of the patterning oligo. (a) Fluorescence image acquired after hybridization. (b) Fluorescence intensities from five spots were quantified and fitted with a Gaussian function, yielding a full width at half maximum (FWHM) of 992 nm. Scale bar, 5 μm.

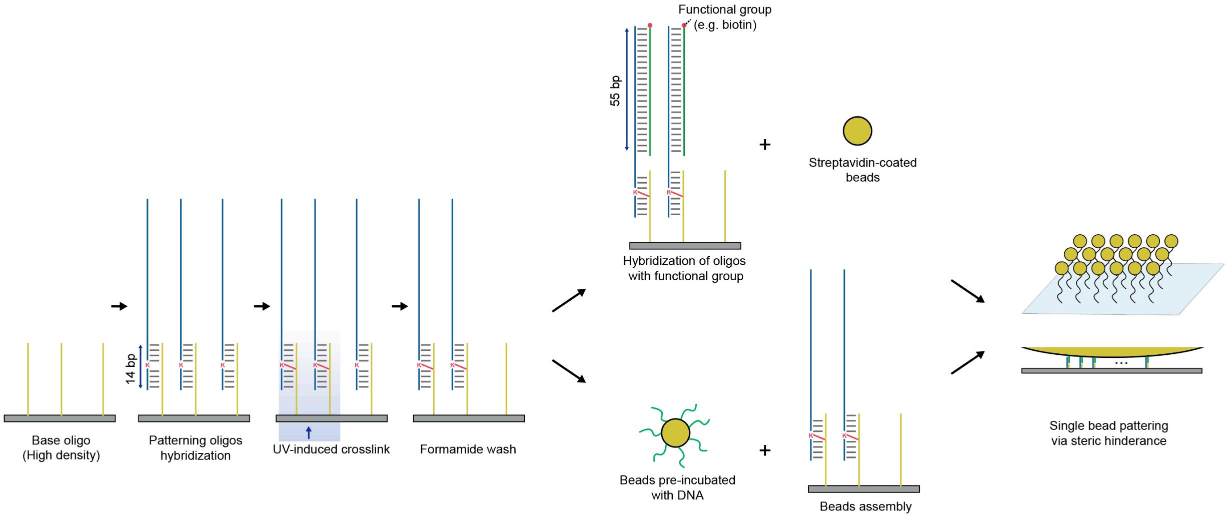

Figure S5. Patterning oligos (blue) serve as linkers between the glass coverslip and the beads. Oligos containing a functional group (e.g. biotin) can hybridize with the patterning oligos to tether beads to the surface. Alternatively, functionalized oligos can first be attached to beads and then subsequently hybridized with the patterning oligos on the surface.

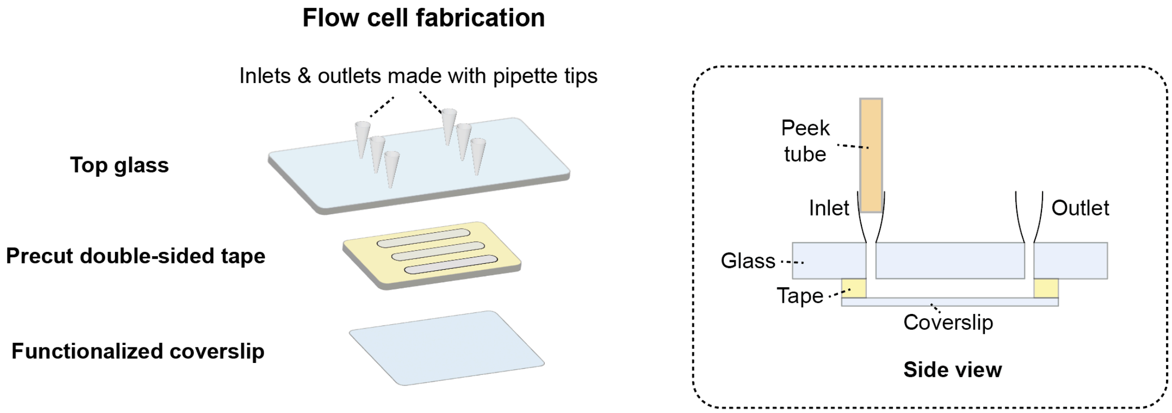

Figure S6. Flow cell fabrication. Inlets and outlets were created by attaching pipette tips to holes drilled in the top glass and sealing them with epoxy. A syringe was connected to the flow cell via PEEK tubing to enable controlled infusion and withdrawal of buffer.

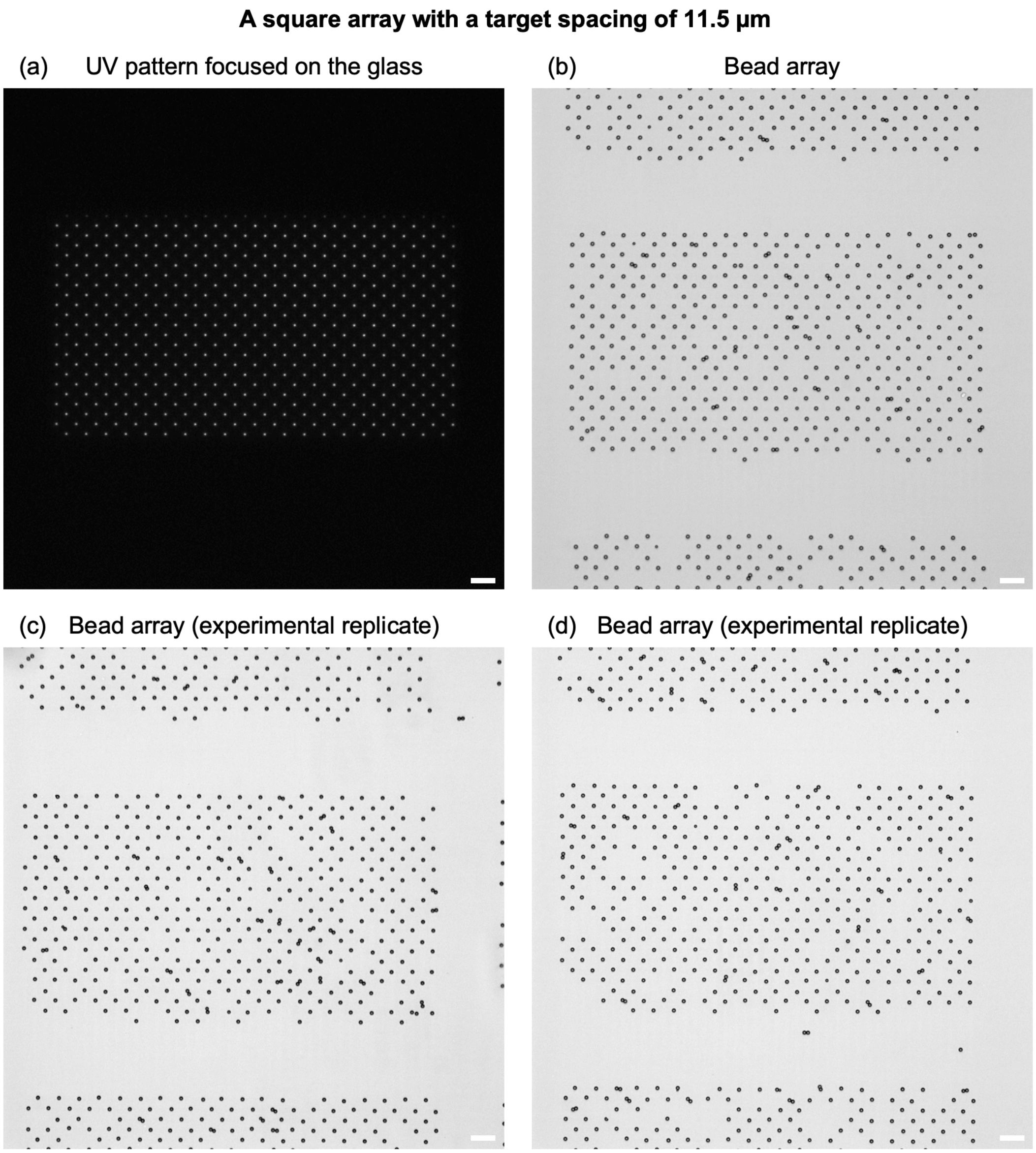

Figure S7. Beads arranged into a square lattice array with a nominal spacing of 11.5 µm. (a) UV illumination pattern projected onto a blank coverslip. (b – d) Beads assembled into square arrays using patterned functional strands; images from three independent experimental replicates are shown. Scale bar, 20 µm.

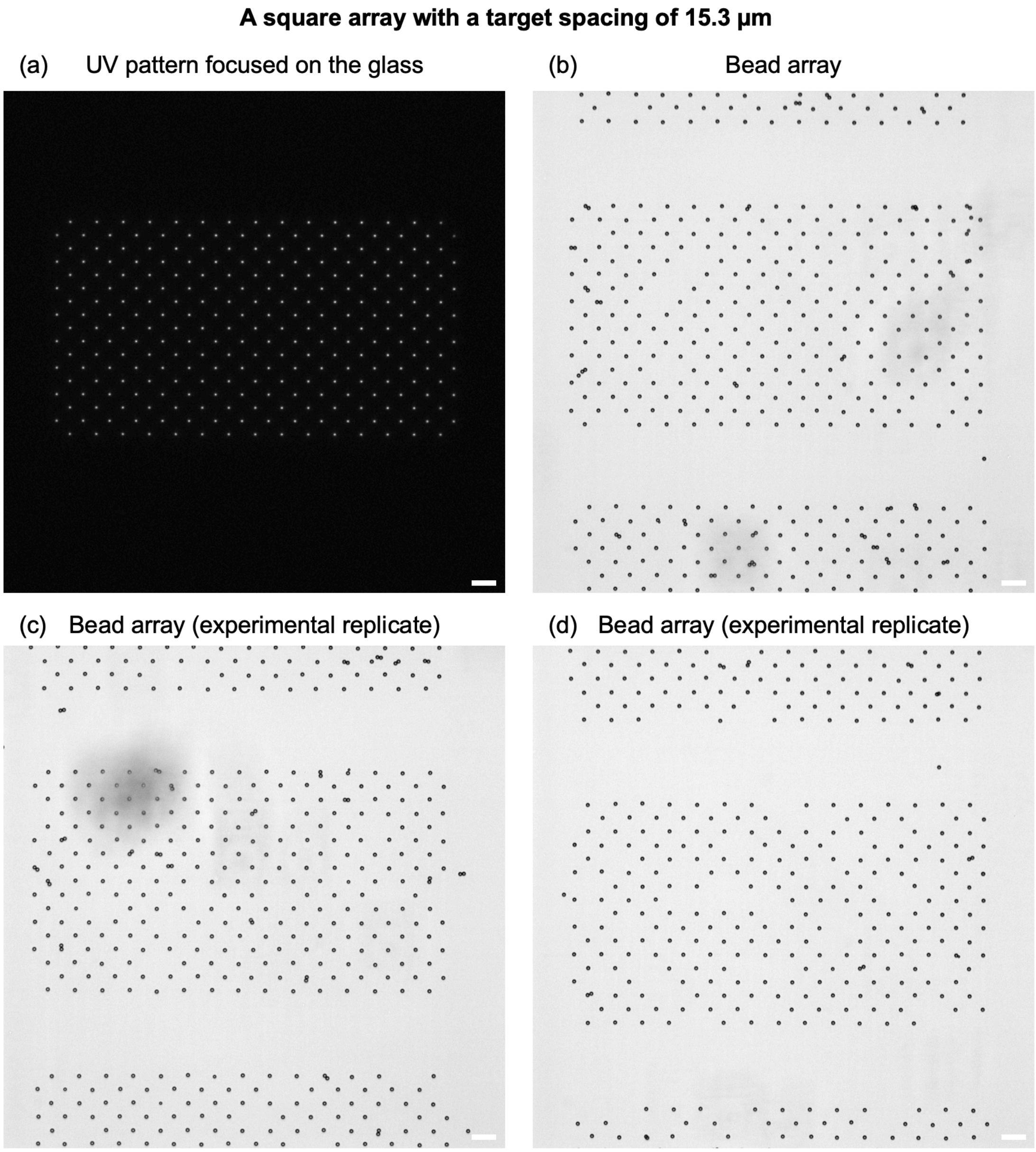

Figure S8. Beads arranged into a square lattice array with a nominal spacing of 15.3 µm. (a) UV illumination pattern projected onto a blank coverslip. (b – d) Beads assembled into square arrays using patterned functional strands; images from three independent experimental replicates are shown. Scale bar, 20 µm.

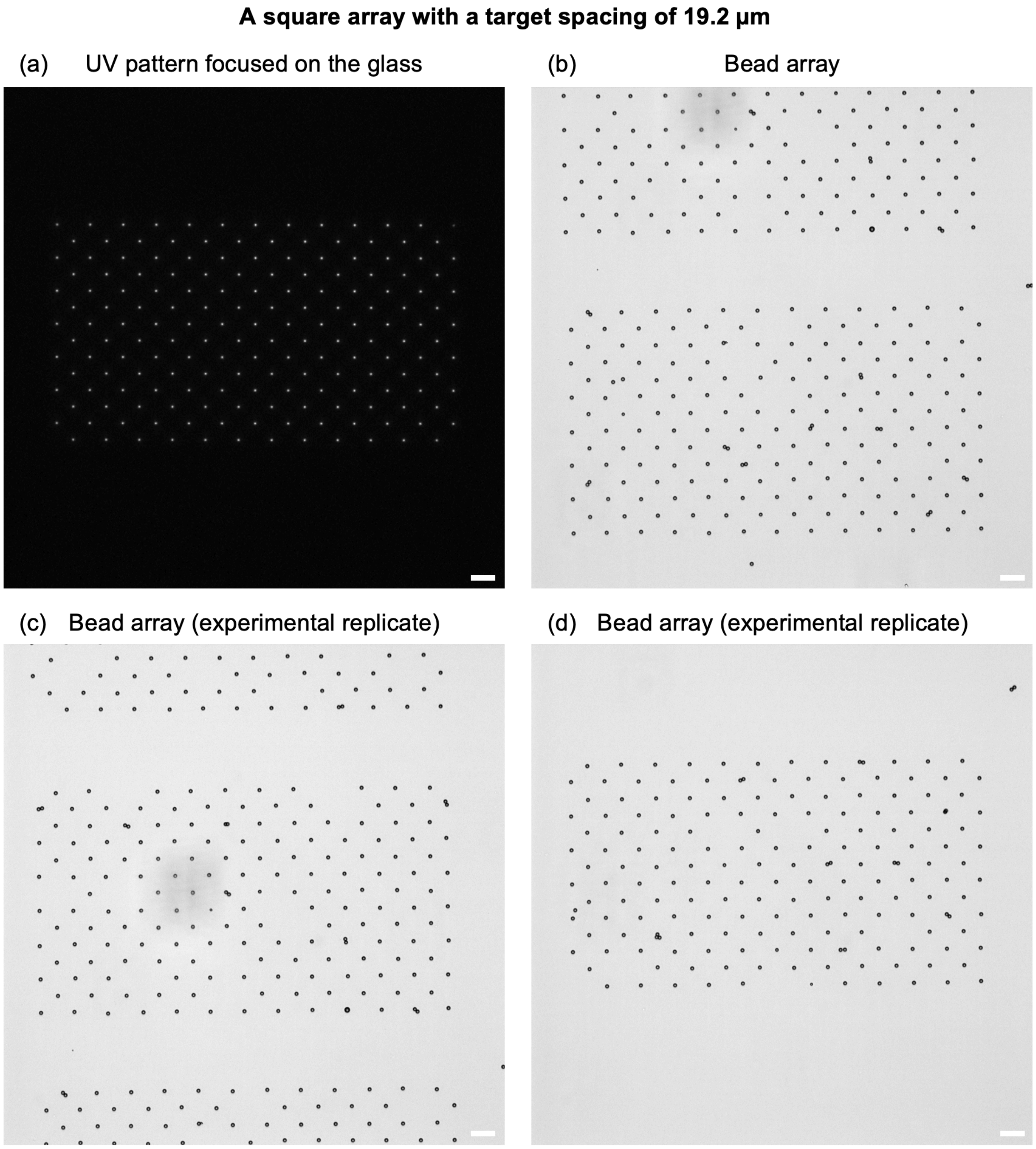

Figure S9. Beads arranged into a square lattice array with a nominal spacing of 19.2 μm. (a) UV illumination pattern projected onto a blank coverslip. (b – d) Beads assembled into square arrays using patterned functional strands; images from three independent experimental replicates are shown. Scale bar, 20 μm.

A hexagonal array with a target spacing of 11.5 μm

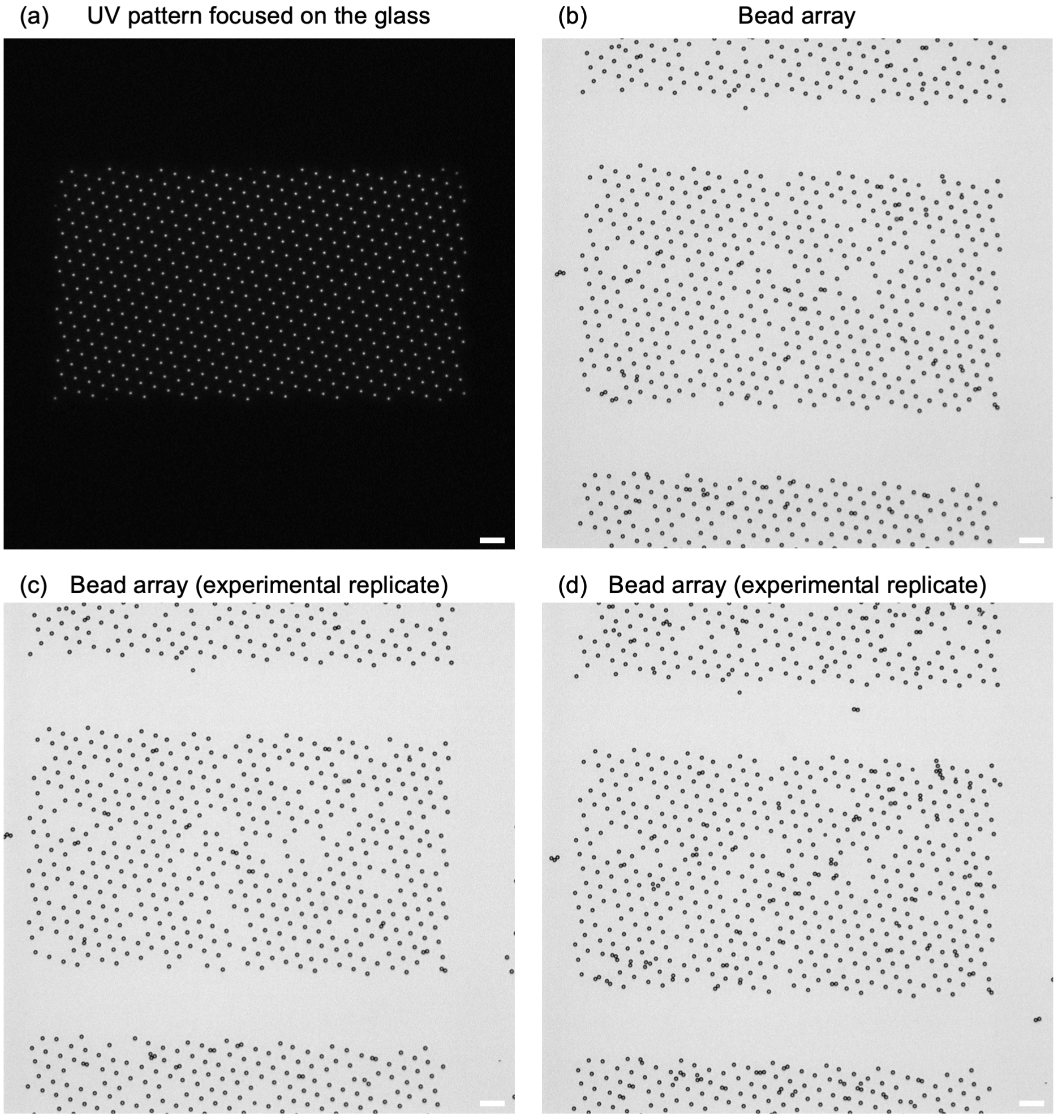

Figure S10. Beads arranged into a hexagonal lattice array lattice with a nominal spacing of 11.5 μm. (a) UV illumination pattern projected onto a blank coverslip. (b – d) Beads assembled into hexagonal arrays using patterned functional strands; images from three independent experimental replicates are shown. Scale bar, 20 μm.

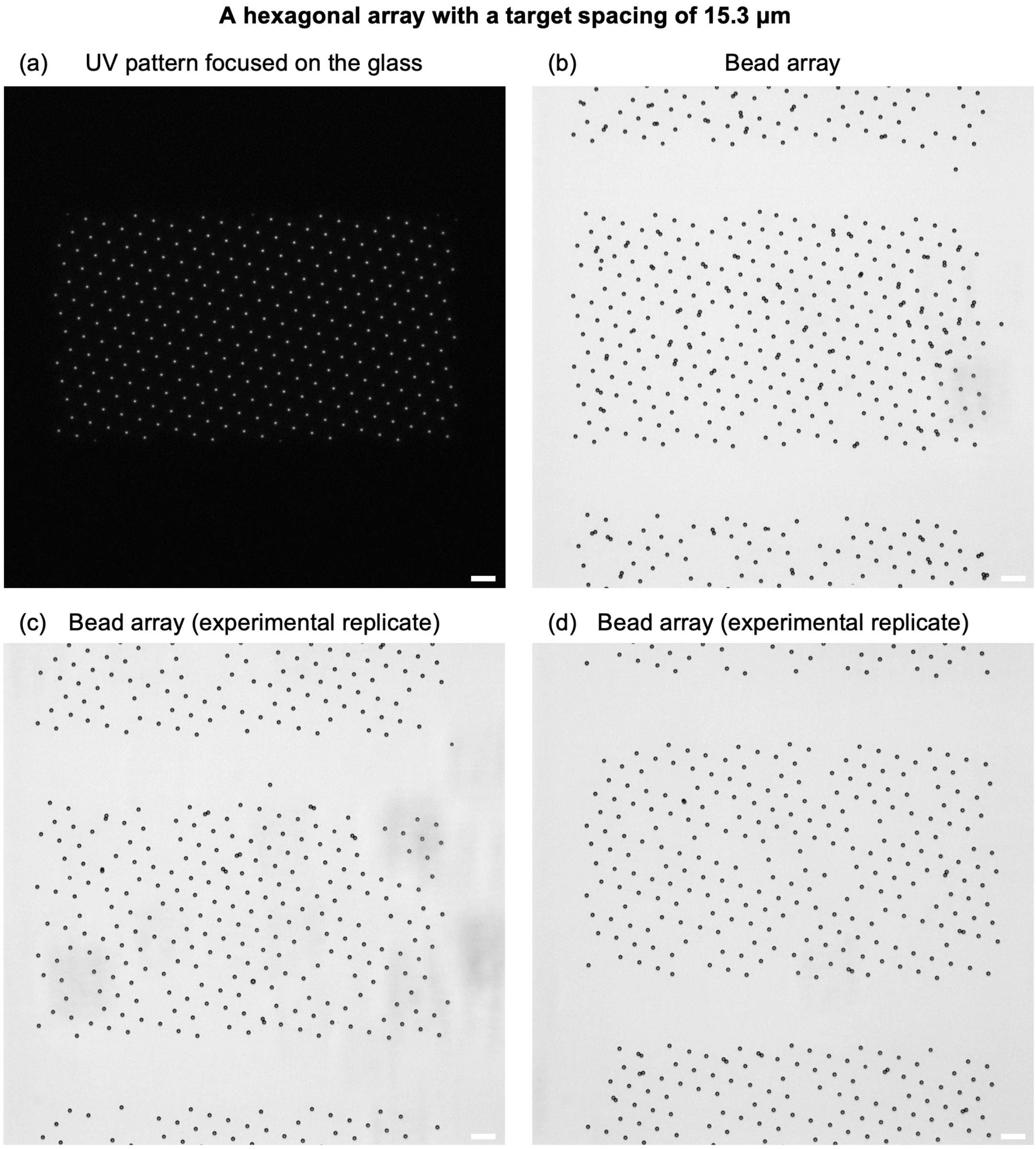

Figure S11. Beads arranged into a hexagonal lattice array with a nominal spacing of 15.3 μm. (a) UV illumination pattern projected onto a blank coverslip. (b – d) Beads assembled into hexagonal arrays using patterned functional strands; images from three independent experimental replicates are shown. Scale bar, 20 μm.

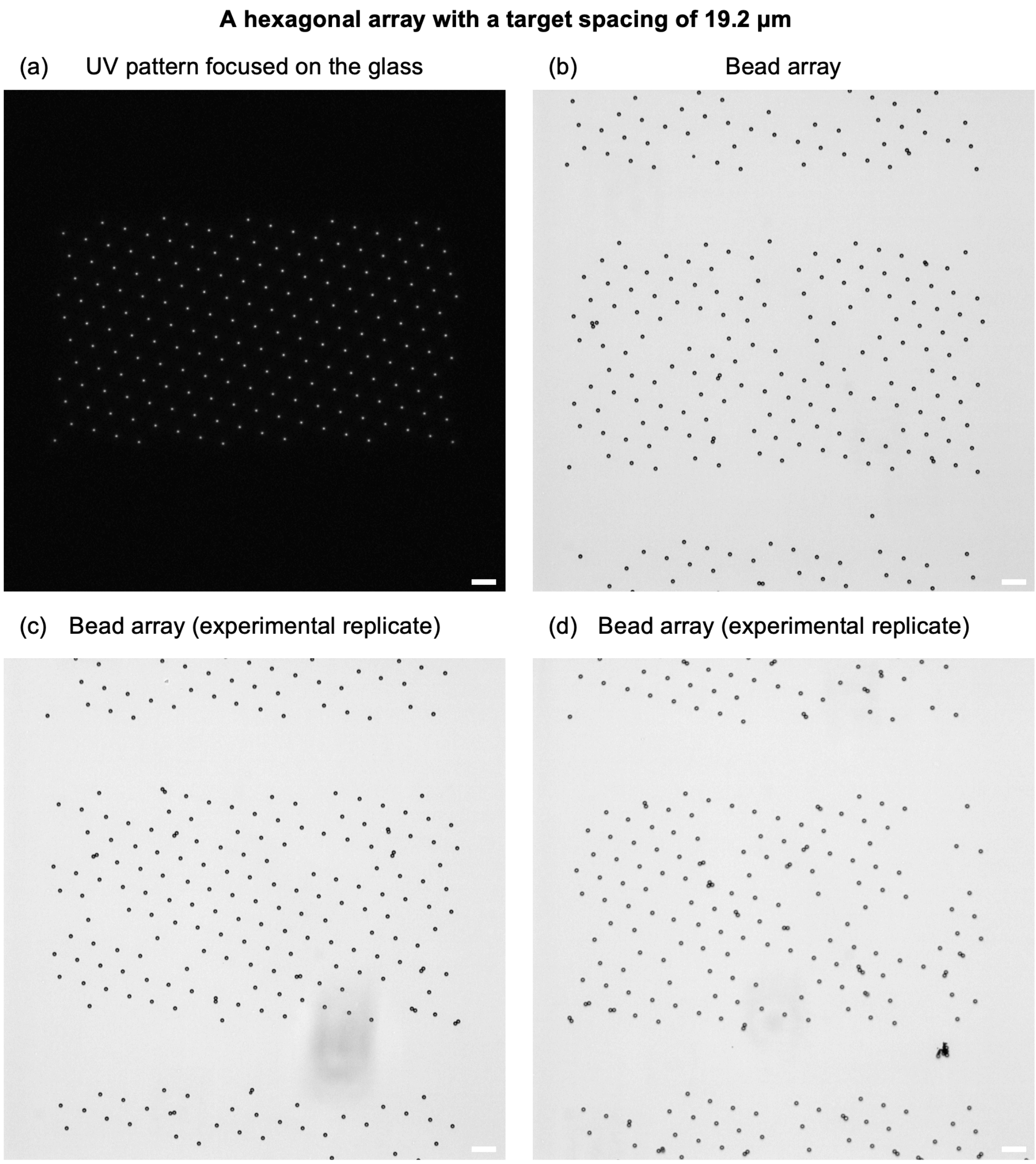

Figure S12. Beads arranged into a hexagonal lattice array with a nominal spacing of 19.2 µm. (a) UV illumination pattern projected onto a blank coverslip. (b – d) Beads assembled into hexagonal arrays using patterned functional strands; images from three independent replicates are shown. Scale bar, 20 µm.

(a) (b)

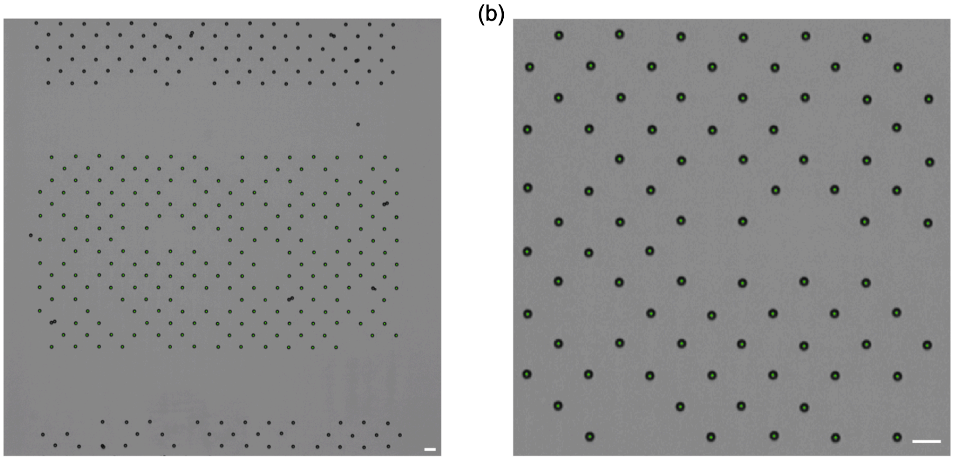

Figure S13. Bead centers (green asterisks) identified using an edge detection algorithm based on the Sobel approximation. (a) Representative image of a bead array arranged in a square lattice with 15.3 μm spacing. Bead dimers were successfully excluded using the algorithm. (b) Magnified view. Scale bar, 10 μm.

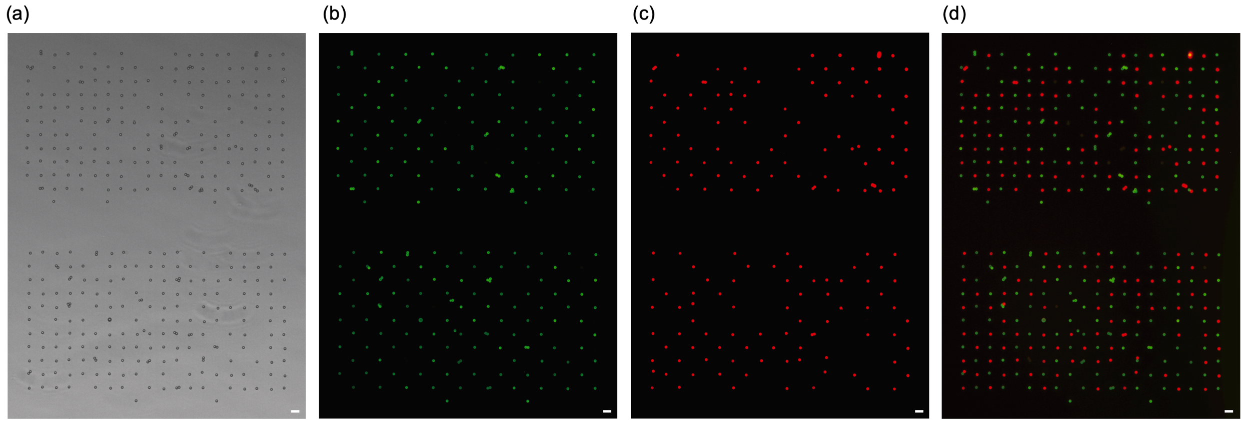

Figure S14. A square bead array is fabricated with two distinct patterning oligos with different functional sequences. From left to right, the images taken with bright field (a), fluorescence channel with a Texas Red filter (b), fluorescence channel with a Cy5 filter (c), and the merged image (d) are shown. Scale bar, 10 µm

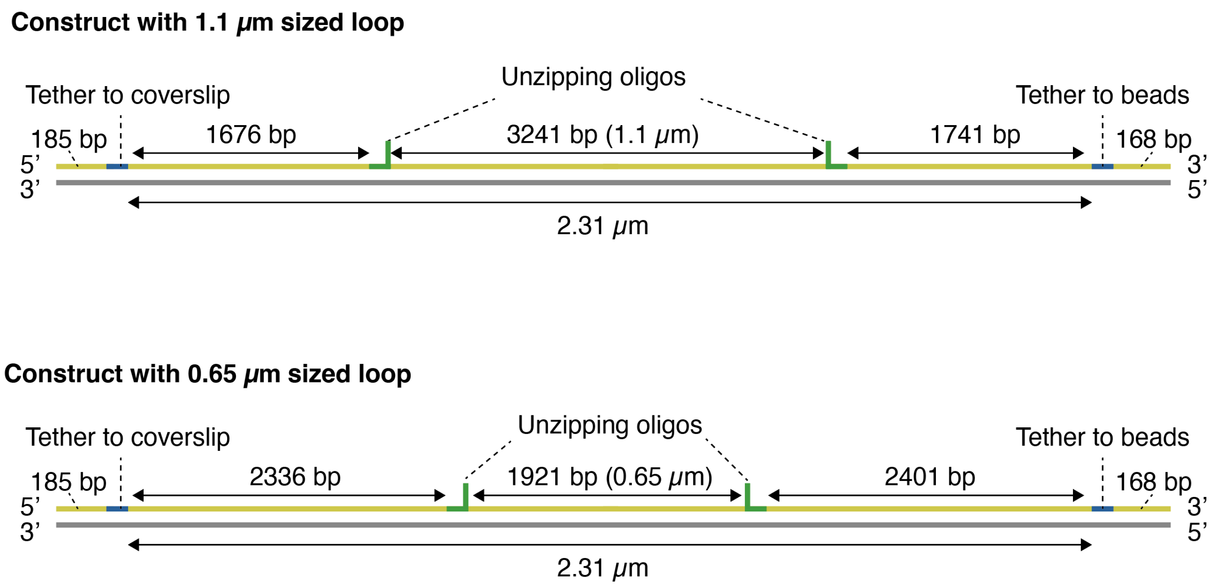

Figure S15. DNA nanoswitch construct design for single-molecule force measurements. Oligo sequences are available in Data S3 and Table S1.

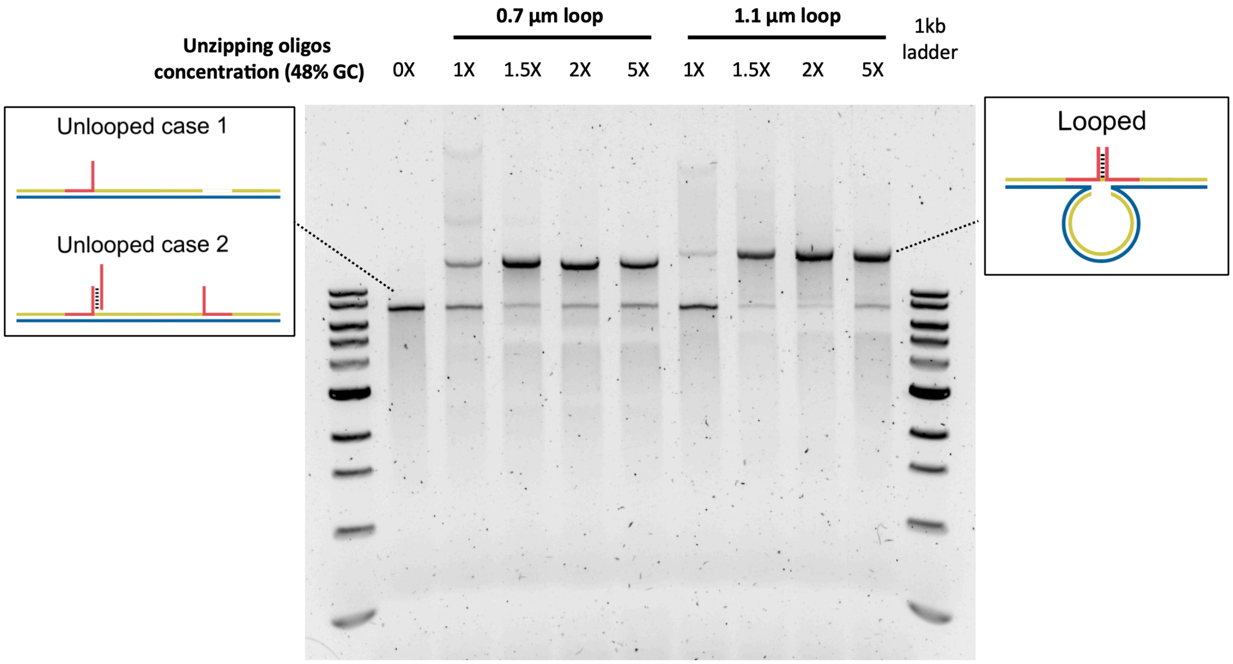

Figure S16. 0.7% Agarose gel electrophoresis result of synthesized constructs. Final concentration of scaffold strand was 1 nM, and that of backbone oligos was 5 nM for one-pot assembly. Unzipping oligos with GC content of 48% were added at a concentration of 1 nM, 1.5 nM, 2 nM, and 5 nM to maximize the ratio of looped constructs and resulting looping ratio were 50.7%, 92.5%, 88.9%, and 80.2% respectively when the size of the loop was 0.7 µm. When the construct with 1.1 µm long loop were fabricated with unzipping oligos concentration of 1 nM, 1.5 nM, 2 nM, and 5 nM, the ratio of looped constructs was 13.2%, 95.2%, 97.5%, and 89.3%.

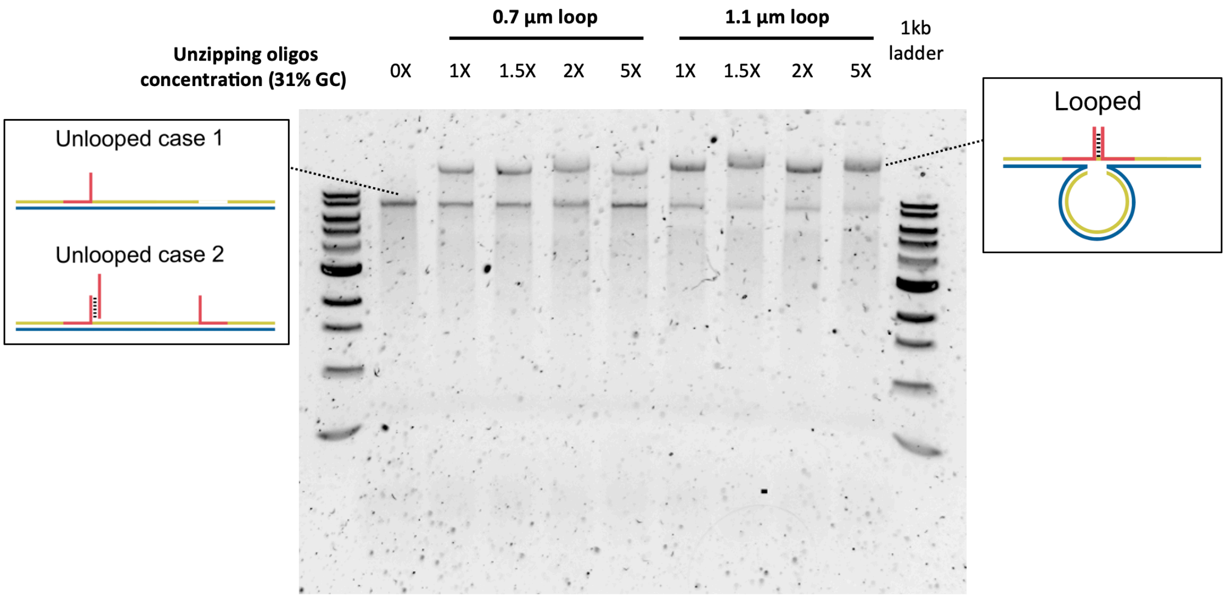

Figure S17. 0.7% Agarose gel electrophoresis result of synthesized constructs. Final concentration of scaffold strand was 1 nM, and that of backbone oligos were 5 nM for one-pot assembly. Unzipping oligos with GC content of 31% were added at a concentration of 1 nM, 1.5 nM, 2 nM, and 5 nM to maximize the ratio of looped constructs and resulting looping ratio was 62%, 59.8%, 51.2%, and 40.9% respectively when the size of the loop was 0.7 µm. When the construct with 1.1 µm long loop were fabricated with unzipping oligos concentration of 1 nM, 1.5 nM, 2 nM, and 5 nM, the ratio of looped constructs was 75.8%, 83.3%, 84.6%, and 88.2%.

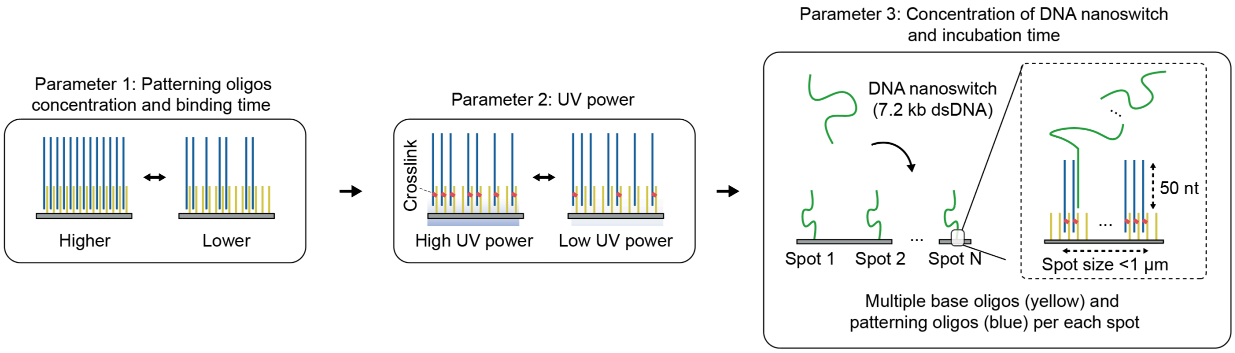

Figure S18. Parameters controlling single-molecule tethering per bead. To reliably achieve single-molecule tethering at each patterned spot, several parameters can be optimized to control the balance between single and multiple tethers. These include: (1) patterning oligo density, adjusted by varying the concentration and surface binding time of patterning oligos; (2) UV exposure, which governs the number of surface-bound patterning oligos covalently crosslinked to the surface; and (3) DNA nanoswitch concentration and incubation time, which influence how many nanoswitch tethers hybridize to patterning oligos within each patterned spot.

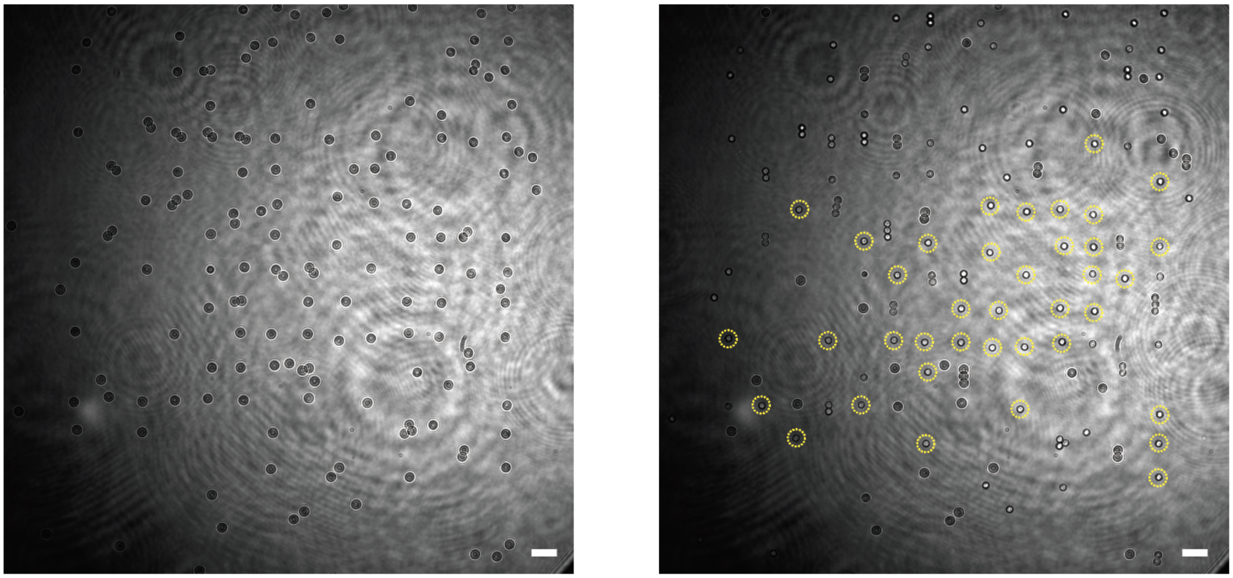

Figure S19. Large field of view image of beads tethered with a 2.3 μm long DNA construct during a magnetic tweezer experiment. The left image was taken without magnetic force, and the right image was taken with a magnetic force of 15 pN. Circled beads indicate those tethered with a single construct.

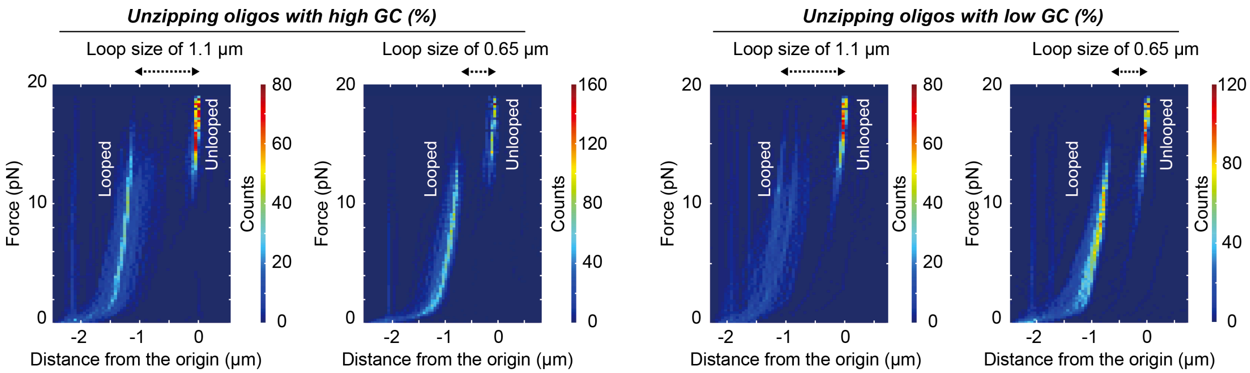

Figure S20. Force-extension curve heatmaps from single-molecule experiments. DNA nanoswitch constructs were prepared using unzipping oligos with two different GC contents (high and low), and two distinct loop sizes (1.1 μm and 0.65 μm). The number of analyzed traces for each condition was: high GC, 1.1μm (N=152), high GC, 0.65 μm (N=274), low GC, 1.1 μm (N=108), and low GC, 0.65 μm (N=276). Force-extension trajectories from multiple beads were pooled and binned, with bin widths of 0.2 pN in force and 50 nm in extension. The reference position (origin) of each bead was set to its position at the highest applied force, as the bead Z-position look-up tables were acquired at maximum force to minimize fluctuations. Black dashed arrows indicate the expected contour lengths of the loops. Colormap represents counts.

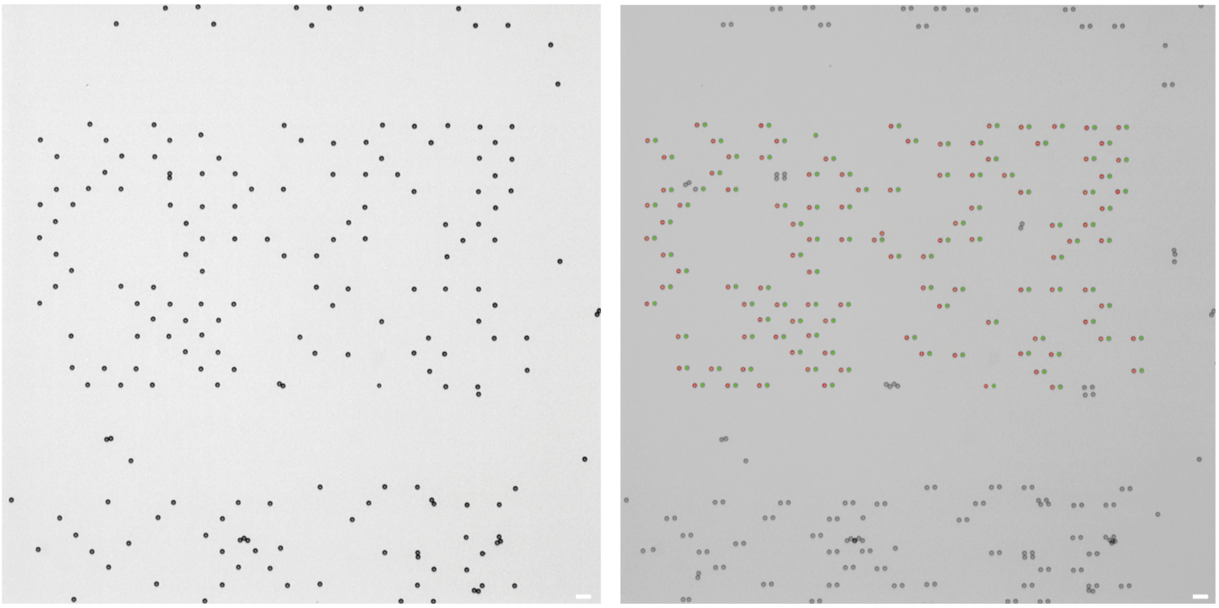

Figure S21. Large field of view image of beads tethered with 2.3 µm long DNA construct. Right image shows merged image from flow infuse and withdrawal, showing the tether lengths. Beads were detected as red circle from the flow infuse, and green asterisk shows detected circle during flow withdrawal.

Base oligo	CTCAGACGAGTCGATTT-[DBCO-PEG13]-3′
Patterning oligo	TCGA[CNVK]TCGTCTGAGTAATGTGTAGGTAAAGATTCAAAAGGGTGAGAAAG GCCGGAGACAGTCAAATCAC
Functional oligo	5′ [Dual-biotin or amine] TTTTTGTGATTTGACTGTCTCCGGCCTTTCTCACCCTTTTGAATCTTTACCTACA
Fluorescent oligo	5′ [ATTO488]TTTTTGTGATTTGACTGTCTCCGGC
Cutting strand	CTACTAATAGTAGTAGCATTAACATCCAATAAATCATACA
48% GC, 1.1 μm loop, unzipping oligo-1	CTGTCCATCACGCAAATTAACCGTTGTAGCAATACTTCTTTGATTAGTAATAACATCACCA CGAATTCTCTGCCTCCCTTTTAACCCTAG
48% GC, 1.1 μm loop, unzipping oligo-2	CTAGGGTTAAAAGGGAGGCAGAGAATTCGTGGTATTAAGAGGCTGAGACTCCTCAAGAGAA GGATTAGGATTAGCGGGGTTTTGCTCAGT
31% GC, 1.1 μm loop, unzipping oligo-1	TCTGTCCATCACGCAAATTAACCGTTGTAGCAATACTTCTTTGATTAGTAATAACATCACC TCAAATATCAAACCCTCAATCAATATCT
31% GC, 1.1 μm loop, unzipping oligo-2	AGATATTGATTGAGGGTTTGATATTTGAGGTATTAAGAGGCTGAGACTCCTCAAGAGAAGG ATTAGGATTAGCGGGGTTTTGCTCAGTA
48% GC, 0.65 μm loop, unzipping oligo-1	TTCGACAACTCGTATTAAATCCTTTGCCCGAACGTTATTAATTTTAAAAGTTTGAGTAACA CGAATTCTCTGCCTCCCTTTTAACCCTAG
48% GC, 0.65 μm loop, unzipping oligo-2	CTAGGGTTAAAAGGGAGGCAGAGAATTCGTGTCAACCGATTGAGGGAGGGAAGGTAAATAT TGACGGAAATTATTCATTAAAGGTGAATT
31% GC, 0.65 μm loop, unzipping oligo-1	ATTCGACAACTCGTATTAAATCCTTTGCCCGAACGTTATTAATTTTAAAAGTTTGAGTAAC TCAAATATCAAACCCTCAATCAATATCT
31% GC, 0.65 μm loop, unzipping oligo-2	AGATATTGATTGAGGGTTTGATATTTGAGTCAACCGATTGAGGGAGGGAAGGTAAATATTG ACGGAAATTATTCATTAAAGGTGAATTA

Table S1. Unzipping oligos sequences for DNA construct.

References

1. Strick, T. R., Croquette, V. & Bensimon, D. Single-molecule analysis of DNA uncoiling by a type II topoisomerase. *Nature* **404**, (2000).
2. Yu, Z. *et al.* A force calibration standard for magnetic tweezers. *Review of Scientific Instruments* **85**, (2014).
3. Te Velthuis, A. J. W., Kerssemakers, J. W. J., Lipfert, J. & Dekker, N. H. Quantitative guidelines for force calibration through spectral analysis of magnetic tweezers data. *Biophys J* **99**, 1292–1302 (2010).
4. Shrestha, P. *et al.* Single-molecule mechanical fingerprinting with DNA nanoswitch calipers. *Nat Nanotechnol* (2021) doi:10.1038/s41565-021-00979-0.
5. Silver, J., Li, Z. & Neuman, K. Tethered-bead, immune sandwich assay. *Biosens Bioelectron* **63**, 117–123 (2015).
